# Phosphoproteomics of short-term hedgehog signaling in human medulloblastoma cells

**DOI:** 10.1186/s12964-020-00591-0

**Published:** 2020-06-23

**Authors:** Tamara Scheidt, Oliver Alka, Humberto Gonczarowska-Jorge, Wolfgang Gruber, Florian Rathje, Margherita Dell’Aica, Marc Rurik, Oliver Kohlbacher, René P. Zahedi, Fritz Aberger, Christian G. Huber

**Affiliations:** 1 Department of Biosciences, Bioanalytical Research Laboratories and Molecular Cancer Research and Tumor Immunology, Cancer Cluster Salzburg, University of Salzburg, Hellbrunner Straße 34, 5020 Salzburg, Austria; 2 Institute for Bioinformatics and Medical Informatics, University of Tübingen, Sand 14, 72076 Tübingen, Germany; 3 Leibniz-Institute of Analytical Sciences- ISAS - e.V, Dortmund, Germany; 4 Present address: CAPES Foundation, Ministry of Education of Brazil, Brasília, DF 70040-020 Brazil; 5 Present address: EVER Valinject GmbH, 4866 Unterach am Attersee, Austria; 6 Biomolecular Interactions, Max Planck Institute for Developmental Biology, Max-Planck-Ring 5, 72076 Tübingen, Germany; 7 Institute for Translational Bioinformatics, University Hospital Tübingen, Hoppe-Seyler-Str. 9, 72076 Tübingen, Germany; 8 Applied Bioinformatics, Center for Bioinformatics, University of Tübingen, Sand 14, 72076 Tübingen, Germany; 9 Gerald Bronfman Department of Oncology, Jewish General Hospital, McGill University, Montreal, Canada; 10 Segal Cancer Proteomics Centre, Lady Davis Institute, Jewish General Hospital, McGill University, Montreal, Canada

**Keywords:** Oncogenic signaling, Hedgehog signaling, Phosphorylation, Phosphoproteomics, Medulloblastoma, Kinases, DAOY cells

## Abstract

**Background:**

Aberrant hedgehog (HH) signaling is implicated in the development of various cancer entities such as medulloblastoma. Activation of GLI transcription factors was revealed as the driving force upon pathway activation. Increased phosphorylation of essential effectors such as Smoothened (SMO) and GLI proteins by kinases including Protein Kinase A, Casein Kinase 1, and Glycogen Synthase Kinase 3 β controls effector activity, stability and processing. However, a deeper and more comprehensive understanding of phosphorylation in the signal transduction remains unclear, particularly during early response processes involved in SMO activation and preceding GLI target gene regulation.

**Methods:**

We applied temporal quantitative phosphoproteomics to reveal phosphorylation dynamics underlying the short-term chemical activation and inhibition of early hedgehog signaling in HH responsive human medulloblastoma cells. Medulloblastoma cells were treated for 5.0 and 15 min with Smoothened Agonist (SAG) to induce and with vismodegib to inhibit the HH pathway.

**Results:**

Our phosphoproteomic profiling resulted in the quantification of 7700 and 10,000 phosphosites after 5.0 and 15 min treatment, respectively. The data suggest a central role of phosphorylation in the regulation of ciliary assembly, trafficking, and signal transduction already after 5.0 min treatment. ERK/MAPK signaling, besides Protein Kinase A signaling and mTOR signaling, were differentially regulated after short-term treatment. Activation of Polo-like Kinase 1 and inhibition of Casein Kinase 2A1 were characteristic for vismodegib treatment, while SAG treatment induced Aurora Kinase A activity. Distinctive phosphorylation of central players of HH signaling such as SMO, SUFU, GLI2 and GLI3 was observed only after 15 min treatment.

**Conclusions:**

This study provides evidence that phosphorylation triggered in response to SMO modulation dictates the localization of hedgehog pathway components within the primary cilium and affects the regulation of the SMO-SUFU-GLI axis. The data are relevant for the development of targeted therapies of HH-associated cancers including sonic HH-type medulloblastoma. A deeper understanding of the mechanisms of action of SMO inhibitors such as vismodegib may lead to the development of compounds causing fewer adverse effects and lower frequencies of drug resistance.

Video Abstract

## Background

The role of protein phosphorylation in the control of cellular behavior has been well appreciated and intensely studied for many years. Phosphorylation is one of the most important post translational modifications (PTMs) and regulated through a well-controlled interplay of kinases and phosphatases to regulate cellular signaling [1]. Thus, protein phosphorylation affects processes such as cellular growth, cell division, and metabolism. Kinases are the driving force of phosphorylation cascades, and kinase dysfunction/dysregulation has been associated with oncogenesis [2–5], as for instance demonstrated for the Epidermal Growth Factor Receptor (EGFR) in various cancers. With its unique potential to monitor thousands of phosphorylation sites in parallel, phosphoproteomics has become one of the key methods to monitor kinase activity in cancer cells and oncogenic pathways [6–8]. One of the first phosphoproteomics studies intensively monitored phosphorylation dynamics in HeLa cells upon treatment with Epidermal Growth Factor (EGF) already in 2006 [9]. Phosphoproteomics has also been used to study various oncogenic signaling pathways such as Ras signaling [10] and PI3K signaling [11]. It has been applied to study lung cancer [12, 13], ovarian cancer [14], neuroblastoma [15, 16], and many other cancer entities.

In vertebrates, the hedgehog (HH) pathway plays a central role in the control of embryonic development, cell proliferation, and survival. The HH pathway is induced by the release of HH ligands such as sonic HH. Upon binding of HH protein to Patched 1 (PTCH-1), the G-protein coupled-like receptor Smoothened (SMO) is released from inhibition by PTCH1, allowing its translocation to and accumulation in the primary cilium. The primary cilium is a microtubule-based surface projection essential for canonical HH signal transduction in vertebrates [17, 18]. After HH pathway activation, SMO induces downstream signaling by inhibition of the central GLI inhibitory molecule SUFU, thereby allowing unprocessed full-length- GLI-transcription factors to translocate into the nucleus to induce HH target gene expression (reviewed in ref. [19]). The movement of hedgehog pathway components into and within the primary cilium is orchestrated by intraflagellar transport proteins as parts of the intraflagellar transport machinery (IFT) [20].

Persistent and irreversible activation of HH signaling has been implicated in the development of various cancers such as basal cell carcinoma and medulloblastoma [21–23]. In order to study phosphorylation dynamics in the HH pathway, Varjosalo et al. screened for kinases implicated in HH signaling by studying the kinome collection [24]. Marumoto et al. used comprehensive phosphoproteomics to reveal a critical role of the HH pathway in osteoblast transitions [25]. Recently, comprehensive phosphoproteomics elucidated Casein Kinase 2 as a key driver of the development of sonic HH-driven human medulloblastoma [26]. Besides Casein Kinase 1, other kinases such as Protein Kinase A and Glykogen Synthase Kinase 3β (GSK3β) are known as antagonistic players in the HH signaling cascade [27, 28]. Phosphorylation of the GLI transcription factors by these kinases promotes their ubiquitination and proteasomal processing or degradation [29]. Kinases were also reported to have dual roles in the pathway regulation. Casein Kinase I α (CSKIα), for instance, contributes to GLI transcription factor processing [30] but has also been implicated in the positive regulation of the HH pathway via phosphorylation of SMO [31]. Furthermore, it has been reported that PKA and GSK3β phosphorylate the inhibitory molecule SUFU, thereby stabilizing its molecular structure and promoting its localization in the primary cilium [32], thus impeding pathway activation.

Phosphatases are the major counterparts of kinases and represent an important class of enzymes involved in the regulation of the HH pathway, and their role in sonic HH signaling has been reviewed recently [33]. However, the knowledge about the function of phosphatases is limited. PP2A has been shown to influence GLI3 localization and activity [34] and to be involved in the dephosphorylation of the ciliary trafficking protein Kif7 [35]. Furthermore, PP4 was shown to be implicated in SMO dephosphorylation [36], and lipid phosphatases such as inositol polyphosphate 5-phosphatase INPP5E are critical for proper ciliary trafficking [37]. PP2C family member phosphatase Wip1, also known as PPM1D, influences the stability and localization of GLI1, indicating a direct modulation of transcriptional activity of GLI1 by dephosphorylation [38].

Despite the examples discussed above, the complex role of protein phosphorylation in the early phase of HH pathway activation and inhibition has not been elucidated so far. It is essential to better understand SMO modifying drugs, particularly in light of resistance development and the severe adverse effects such as muscle spasms caused by a number of, but not all, SMO inhibitors [39]. Hence, our study aims at the extensive analysis of phosphorylation patterns after short-term activation and inhibition of the HH pathway in human medulloblastoma cells using comprehensive, HPLC-MS/MS-based phosphoproteome analysis upon enrichment of phosphopeptides. As a cellular model, we used the HH responsive medulloblastoma cell line DAOY [40] to analyze phosphorylation changes in early HH signaling. Smoothened Agonist (SAG) [41] was utilized to activate the HH pathway in vitro, whereas vismodegib (Vismo) – an FDA-approved drug for the treatment of advanced and metastatic basal cell carcinoma [42] - served as inhibitor of the HH pathway. Taking advantage of comprehensive databases of biological pathways and interactions, as well as their function in cancer, such as the Ingenuity Database, we shed light on the complex global phosphorylation patterns in the context of activation and inhibition of the HH pathway in human cancer cells.

## Methods

### Experimental design and statistical rationale

**Fig. 1:**
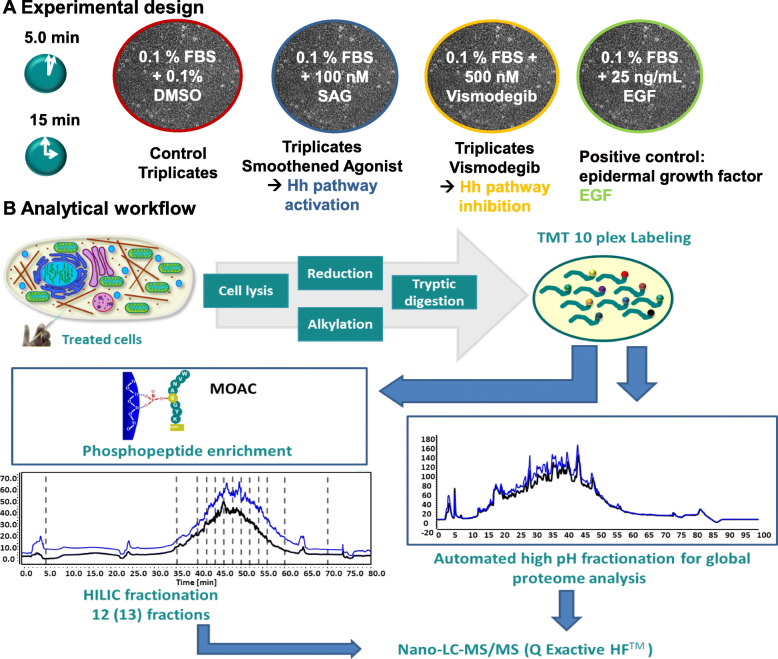
Scheme of experimental design (**a**) and analytical workflow. **a** Three biological replicates of human medulloblastoma cells were treated with DMSO, SAG, vismodegib or EGF in starving medium for 5.0 and 15 min. **b** Analytical workflow: After treatment, sample preparation, and TMT 10-plex labeling, peptides of each time point and treatment were pooled. Aliquots were utilized for pH 8 fractionation and deep proteome profiling or phosphopeptide enrichment by metal oxide affinity chromatography. Measurements of peptides and phosphopeptides were conducted with reversed-phase HPLC coupled to high-resolution mass spectrometry

In order to systematically study the role of phosphorylation in hedgehog-driven cancer signaling, we used the human medulloblastoma cell line DAOY for our studies, which we have previously shown to allow manipulation of canonical pathway activity by (partial) agonist and antagonist treatment [40]. We confirmed the HH responsiveness of DAOY cells by treatment with SAG and vismodegib followed by qPCR analysis of the HH target genes GLI1, HHIP and PTCH1 (Figure S-1). To demonstrate comparable activities of natural and synthetic HH pathway inducers (i.e. natural Sonic Hh protein and the synthetic Smoothened agonist compound SAG, respectively), we performed time course analysis of HH target gene expression by qPCR (Figure S-2). For global phosphoproteomics, we performed short-term treatment of DAOY cells with SAG and vismodegib. For both time points, i.e. 5.0 and 15 min, we stimulated the cells with DMSO, SAG and vismodegib in biological triplicates (Fig. 1a). We used DMSO treatment for 5.0 and 15 min, respectively, in biological triplicates as controls, thus allowing the analysis of control, SAG and vismodegib treatment for a single time point within a single TMT 10-plex experiment. This ensures statistical power and comparability independent of instrumental variability. EGF treatment, known to induce extensive phosphorylation, served as (a single) positive control, by mixing DAOY cells treated for 5.0 and 15 min to a single control. This sample was used as common 10th channel in both TMT 10-plex sample sets (5.0 min and 15 min) to allow the quantitative comparison of the different time points of treatment. The labelling scheme for each TMT 10-plex sample is provided in Table 1.

**Table 1:** TMT Labeling Scheme for Sample 1^a^ and Sample 2^b^

Sample 1^a^	Sample 2^b^	TMT label
DMSO_5.0 min	DMSO_15min	126
DMSO_5.0 min	DMSO_15min	127n
DMSO_5.0 min	DMSO_15min	127c
SAG_5.0 min	SAG_15min	128n
SAG_5.0 min	SAG_15min	128c
SAG_5.0 min	SAG_15min	129n
Vismo_5.0 min	Vismo_15min	129c
Vismo_5.0 min	Vismo_15min	130n
Vismo_5.0 min	Vismo_15min	130c
EGF 5.0 + 15 min	EGF 5.0 + 15 min	131

^a^5.0 min treatment; ^b^15 min treatment

#### Scheme of experimental design (A) and analytical workflow

(A) Three biological replicates of human medulloblastoma cells were treated with DMSO, SAG, vismodegib or EGF in starving medium. (B) Analytical workflow: After treatment, sample preparation, and TMT 10-plex labeling, peptides of each time point and treatment were pooled. Aliquots were utilized for pH 8 fractionation and deep proteome profiling or phosphopeptide enrichment by metal oxide affinity chromatography. Measurements of peptides and phosphopeptides were conducted with reversed-phase HPLC coupled to high-resolution mass spectrometry.

After cell lysis, reduction and alkylation, tryptic digestion, and TMT labeling for relative quantification, phosphopeptides were enriched by metal oxide affinity chromatography (MOAC) using titanium dioxide (TiO_2_) beads. Following enrichment, phosphopeptides were fractionated using hydrophilic interaction liquid chromatography (HILIC) prior to nano-HPLC-MS/MS analysis for detection and relative quantification of phosphopeptides (Fig. 1b). In parallel, deep proteome profiling was performed with 50 μg of the sample using high pH fractionation to normalize the phosphoproteome data and to detect potential changes in protein abundance. For each phosphopeptide (TiO_2_) or protein (global), the average of the three normalized intensity values of each treatment or control was used to calculate the ratio of each particular treatment versus control. All three biological replicates were considered for ratio calculation. To determine *p*-values and statistical significance of more than 7000 phosphopeptides in an unbiased manner, LIMMA (Linear Models for Microarray) statistical testing was applied.

### Chemicals and samples

Hyperconfluent HH responsive DAOY cells [40] (ATCC: HTB-186 American Type Culture Collection, ATCC, Manassas, VA, USA) pre-starved for 24 h in serum-reduced MEM medium containing 0.1% (v/v) FBS were treated with 0.10% (v/v) DMSO, 100 nM Smoothened Agonist (SAG, Sigma-Aldrich, Steinheim, Germany), 500 nM vismodegib (vismodegib, LC Laboratories, Woburn, USA), or 25 ng/mL Epidermal Growth Factor (EGF, Sigma-Aldrich, Steinheim, Germany) for 5.0 and 15 min, respectively, in triplicates. After treatment, the cells were washed with PBS prior to lysis in a solution containing 1.0% SDS (w/v), 50 mM Tris-HCl pH 7.80, 150 mM NaCl, supplemented with protease inhibitor cocktail Complete Mini and phosphatase inhibitor cocktail PhosSTOP (Roche, Basel, Switzerland). To reduce viscosity and remove nucleic acid, samples were incubated with 5.0 μL benzonase nuclease (25 units/μl) (Merck, Darmstadt, Germany) for 30 min at 37 °C, followed by centrifugation at 18,000 g for 30 min at 4.0 °C. Pellets were discarded. To determine protein concentration, a bicinchoninic acid (BCA) assay (Thermo Scientific, USA) was performed according to the manufacturer’s protocol. Cysteines were reduced with DTT (10 mM final concentration, Roche Diagnostics, Mannheim, Germany) for 30 min at 56 °C and alkylated with iodoacetamide (30 mM final concentration, Sigma Aldrich, Steinheim, Germany) for 20 min at room temperature in the dark. For each sample, 150 μg of protein were loaded onto a 30 kDa molecular weight cut-off spin filter (Pall Nanosep®, Sigma-Aldrich, Germany) for filter-aided sample preparation [43, 44] with a few modifications. Protein digestion occurred in 50 mM triethylammonium bicarbonate (TEAB, Sigma Aldrich, Steinheim, Germany), 2.0 mM CaCl_2_ (Merck, Darmstadt, Germany), 200 mM guanidine hydrochloride and trypsin (ratio 1:20 enzyme:protein, w/w) (Promega, USA) for 14 h at 37 °C. Later, spin filters were transferred to new 2 mL protein LoBind Eppendorf tubes (Sigma Aldrich, Steinheim, Germany), centrifuged at 13,500 g for 25 min, and followed by two other 15 min centrifugation steps with the addition of 100 μL 50 mM TEAB and 50 μL water on top of the membrane. To control digestion efficiency, 1.0 μg of each digest was injected into a monolithic HPLC system as described in Burkhart et al. [45]. All samples were dried under vacuum.

### TMT 10-plex labeling

Samples were individually solubilized in 100 mM TEAB buffer and incubated with 0.80 mg TMT label (Thermo Fischer Scientific, USA) (dissolved in 41 μL acetonitrile) for 60 min at 25 °C with slight agitation. Reaction was stopped upon incubating for 15 min at 25 °C with 0.27% hydroxylamine (v/v). Samples were pooled together in a ratio of 1:1 and dried under vacuum.

### Sample desalting

TMT pooled samples were solubilized in 300 μL 0.10% aqueous TFA and desalted with SPEC C18 AR 15 mg cartridge (Agilent, Germany). The cartridge was first washed with 400 μL 100% acetonitrile (ACN), equilibrated twice with 400 μL 0.10% (v/v) TFA, loaded twice with TMT sample, washed twice with 400 μL 0.10% (v/v) aqueous TFA, and the sample was eluted twice with 200 μL 70% (v/v) ACN into a protein LoBind Eppendorf tube (Sigma-Aldrich, Steinheim, Germany). After vortexing, 50 μg of each pool were removed for high pH reversed-phase fractionation. Samples were dried under vacuum.

### High-pH reversed-phase fractionation

The aliquots were solubilized in 15 μL10 mM ammonium acetate, pH 8.0 (buffer A) and fractionated on an HPLC System (Ultimate 3000, Thermo Scientific). Peptides were separated on a 150 mm × 1.0 mm i.d. C18 column (ZORBAX 300SB, Agilent Technologies, Germany) with a 55 min gradient ranging from 2.5 to 42% ACN in 10 mM ammonium acetate, pH 8.0, at a flow rate of 12.5 μL/min. In total, 20 fractions were collected in 60 s intervals in concatenation mode. Each fraction was dried under vacuum and resuspended in 15 μL of 0.10% (v/v) aqueous TFA for nano-HPLC–MS/MS analysis.

### Phosphopeptide enrichment and HILIC fractionation

TMT pooled samples were solubilized in 80% (v/v) ACN, 5.0% (v/v) TFA and 1.0 M glycolic acid. Based on Engholm-Keller’s et al. [46] protocol, phosphopeptide enrichment was applied as described in Gonczarowska-Jorge et al. [47]. Phosphopeptides were HILIC fractionated on an HPLC system (Ultimate 3000 nano RSLC, Thermo Scientific) coupled with a self-made 150 mm × 0.250 mm i.d. TSKgel Amide-80 (Tosoh, Japan) column. After injection, sample was loaded for 20 min in 98% ACN, 0.10% TFA (eluent A) and fractionated in 40 min gradient ranging from 1 to 35% eluent B (0.10% TFA). At 10% eluent B, flow rate decreased linearly from 3.0 μL/min to 2.5 μL/min at 40% eluent B. A total of 12 fractions were collected for each sample. Each fraction was dried under vacuum and resuspended in 15 μL of 0.10% aqueous TFA for nano-HPLC–MS/MS analysis.

### Nano-HPLC-MS/MS analysis

Samples were analyzed using a nano HPLC system (Ultimate 3000 nano RSLC, Thermo Scientific) coupled to a quadrupole-Orbitrap mass spectrometer (Q Exactive HF, Thermo Scientific) in data dependent acquisition mode. All samples were preconcentrated on a 20 mm × 75 μm i.d. trapping column (C18 Acclaim Pepmap, Thermo Scientific, Germering, Germany) in 0.1% aqueous TFA for 10 min using a flow rate of 20 μL/min, followed by separation in a 50 cm × 75 μm i.d. separation column (Acclaim Pepmap C18, Thermo Scientific) at a flow rate of 250 nL/min using a binary gradient (A: 0.10% aqueous formic acid, B: 84% acetonitrile, 0.1% formic acid) as indicated in Table 1. MS survey scans were acquired in the Orbitrap from m/z 300 to 1500 at a resolution of 60,000 using the polysiloxane ion at 371.1012 m/z as lock mass [48]. The AGC target value was 2 × 10^5^ and the maximum injection time 120 ms. For MS/MS, precursors were selected using the top 15 ions with 30 s dynamic exclusion. Peptides were fragmented using higher-energy collisional dissociation (HCD) at 33% relative collision energy, and fragment ion spectra were acquired in the Orbitrap with a resolution of 60,000, an AGC target value of 2 × 10^5^ ions, a maximum injection time of 200 ms, and an isolation window of 0.4 m/z for the high pH fractions and isolation window of 0.8 m/z for the HILIC fractions (phosphoproteome). A Table with an overview of applied gradients is added in the supplementary section in Table S-1.

### Data analysis

MS raw files were converted to the open standard format mzML and centroided using msconvert (3.0.11781). Further processing was performed using the KNIME Analytics platform (Version 3.7.0) [49] and OpenMS 2.4.0 [50, 51].

Identification was performed using the MSGFPlusAdapter with MS-GF+ [52](v2018.01.30). A Swiss-Prot human (# of entries: 20410, downloaded 17th of October 2018), cRAP (# of entries: 116) decoy database was created using the DecoyDatabase tool (# of entries: 41050). For database search, enzyme specificity was set to trypsin; Carbamidomethyl (C) (+ 57.021 Da), TMT6plex (N-term, K) (+ 229.163 Da) were set as fixed modifications. Oxidation (M) (+ 15.995 Da) was set as variable modification. For the HILIC fractions, phosphorylation of Ser, Thr, and Tyr (+ 79.966 Da) was included in the variable modifications. MS and MS/MS tolerances were set to 10 ppm and 0.02 Da, respectively. Afterwards, target decoy annotation was added, posterior error probability estimation was performed using percolator (v 3.02.0) [53]. Peptides with a false discovery rate below 0.01 were retained. For the dataset with phospho modifications LuciphorAdapter was used to run LuciPHOr2 (v 1.2014Oct10) [54] for phosphosite localization. Here, phosphopeptides with a False Localization Rate (FLR) smaller 0.05 were considered for further analysis. Quantification was performed using the IsobaricAnalyzer with the TMT10plex method and the correction matrix corresponding to the used chemicals. Afterwards, identification and quantitative information were mapped, unannotated and unassigned identifications filtered and exported in the mztab format.

Further location of the phosphosite position in the protein was assessed by extracting the position based on the annotated protein in the fasta database and the information of the peptide phosphosite localization.

For the proteomics dataset, the peptide level analysis was performed as described above, apart from phospho modifications and phosphosite scoring. Additionally, the unfiltered identifications were used for protein inference performed with the FidoAdapter using Fido (2012) [55]. Protein level quantification was performed using the Fido results with a protein FDR < 0.01.

The normalization of the phosphoproteome was performed as described by Shema et al. [56]. Median normalization of each channel was performed for the proteome. In order to facilitate reproduction of the computational analysis workflows and R scripts are included in the supplementary material.

LIMMA statistical testing (R package limma v. 3.36.5) [57] was applied on proteome normalized log2 intensity values of the phosphoproteomics dataset and used to generate volcano plots using R version 3.5.1. Scripts are attached in the online supplementary and all applied R packages with corresponding versions and citations are provided in Table S-2. Kinase Substrate Enrichment [58] was performed using KSEA App (casecpb.shinyapps.io/ksea/) [59] using the R package “KSEAapp” on CRAN using R version 3.5.1. Pathway enrichment was performed using Ingenuity pathway Analysis IPA (QIAGEN Inc., https://apps.ingenuity.com/).

## Results and discussion

### Comprehensive phosphopeptide profiling detects almost 10.000 phosphosites in stimulated medulloblastoma cells

Following the analytical strategy described above, we identified and quantified a total of 7696 highly confident phosphopeptides after 5.0 min and 9976 phosphopeptides after 15 min treatment with a false localization rate (FLR) below 0.05. We further quantified 7547 protein groups after 5.0 min and 7763 protein groups after 15 min using extensive pH 8 fractionation. As expected, the changes in the global proteome after short-term treatment were negligible. We found a normal and narrow distribution of the ratios SAG/DMSO, Vismo/DMSO and SAG/Vismo, similar to the distribution of the positive control EGF as depicted in Figure S-3. Interestingly, the total number of identified phosphosites was higher after 15 min treatment, whereas the total number of identified protein groups was not increased after 15 min treatment. This can be due to technical variation in the sample preparation for the phosphoproteomics analysis or more phosphorylation events upon 15 min stimulation due to the activation of downstream phosphorylation cascades.

**Fig. 2:**
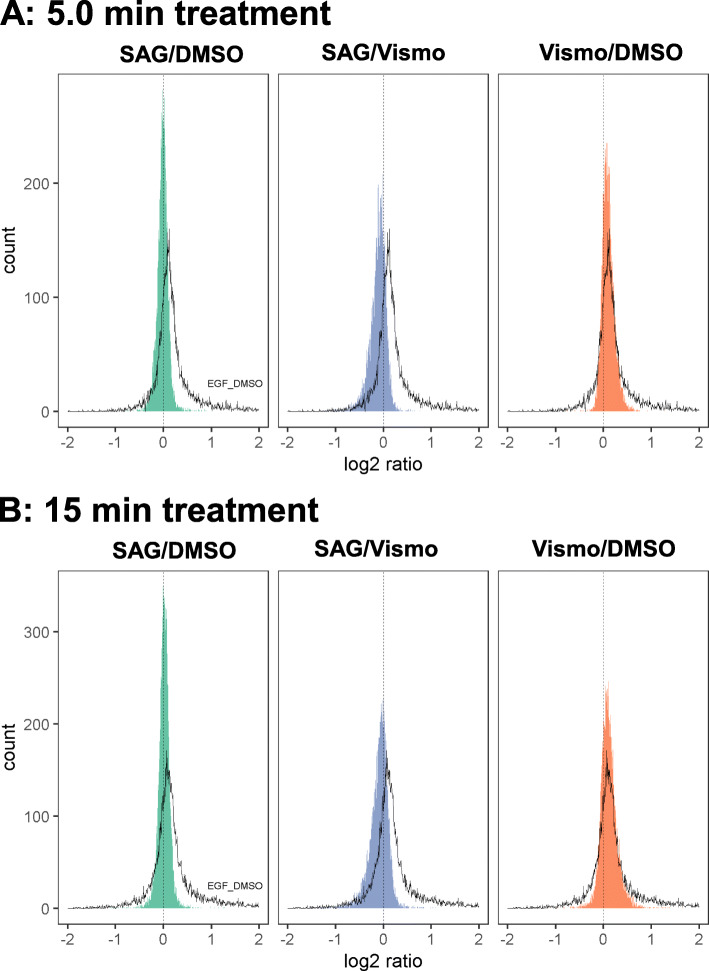
Distribution of proteome normalized ratios of phosphopeptides identified after 5.0 min treatment (**a**) and 15 min treatment (**b**). Intensity values of each channel of the phosphoproteome were normalized by correction factors derived from respective channels of the measured proteome. Ratios were determined by the mean of the treatment SAG or vismodegib (*N* = 3) divided by the mean of DMSO (*N* = 3) of the respective time point. SAG/DMSO (green graph) represents the distribution of the phosphopeptide ratios of SAG treatment compared with DMSO treatment, whereas Vismo/DMSO (orange graph) describes the distribution of phosphopeptide ratio of vismodegib treatment compared to control (DMSO) treatment. The ratio was determined by division of the proteome-normalized intensity by the mean of DMSO treatment. SAG/Vismo (blue graph) describes the phosphopeptide ratio generated by division of SAG/DMSO by Vismo/DMSO

Strikingly, there were several distinctive features in the distributions of the phosphopeptide ratios SAG/DMSO, Vismo/DMSO and EGF/DMSO. Figure 2a illustrates the distribution of phosphopeptide ratios determined from proteome-normalized data for 5.0 min treatment and Fig. 2b for 15 min, respectively. The illustration of the distribution of the phosphopeptide ratios of the pooled EGF treated cells to DMSO treatment (EGF/DMSO) was taken as a reference distribution (black line). EGF is known to induce various phosphorylation events in the DAOY cell line by activation of EGFR signaling [60]. Hence, the reference distribution is skewed to positive ratios. In contrast, only minor changes in the total phosphoproteome were found after SAG treatment. The distribution of SAG/DMSO (green graph) after 5.0 min is quite narrow and almost normally distributed. The broader distribution of vismodegib (Vismo/DMSO) (orange graph) is skewed to positive ratios similar to the reference distribution of EGF/DMSO. This indicates an upregulation of phosphorylation events after 5.0 min of vismodegib treatment. These effects are maintained after 15 min treatment. The distribution of the ratio of Vismo/DMSO is even broader and more prominently skewed to positive ratios. This indicates that in particular inhibition of the Hedgehog pathway simulated by vismodegib treatment in DAOY cells is driven by a global increase in phosphorylation.

### Ingenuity pathway analysis reveals differences in regulation of various cancer related pathways after 5.0 min treatment

**Fig. 3:**
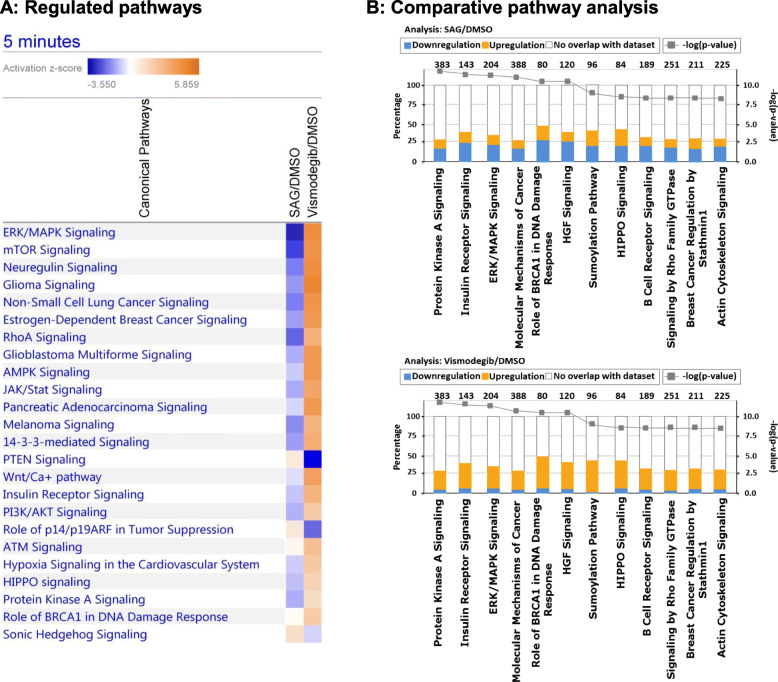
Ingenuity pathway analysis of all phosphopeptides identified after 5.0 min. **a** Comparative analysis of regulated pathways after SAG and vismodegib treatment. Pathways were manually filtered for cancer relevance and the log10 *p*-value cut-off was set to 1.3. Activated pathways are shown in orange, inhibited pathways in blue. **b** Top 12 significantly enriched pathways after 5.0 min SAG (upper bar chart) and vismodegib treatment (lower bar chart). The total number of phosphoproteins which are assigned to a particular pathway are displayed on top of the particular bar, share of those phosphoproteins identified in the experiment is represented by the primary x-axis, while the –log *p*-value determined by Fisher’s exact testing is represented by the secondary y-axis. The proportion of upregulated phosphoproteins is shown in orange, downregulation is indicated by blue bars

Ingenuity pathway analysis (IPA) was performed with all quantified phosphopeptides for both time points. All proteome-normalized phosphopeptide ratios of respective treatment relative to DMSO as control treatment were uploaded and analyzed using the phosphorylation analysis tool in the IPA software platform. Notably, differences in global phosphorylation dynamics between activation and inhibition of the HH pathway were more pronounced after the short-time treatment time point of 5.0 min as depicted in Fig. 3.

Protein phosphorylation is a fast and highly dynamic process, which can transmit intracellular signaling within minutes and even seconds [9, 61, 62]. Ingenuity pathway analysis of our data revealed various cancer-related pathways as differentially regulated already after 5.0 min of vismodegib or SAG treatment (Fig. 3a). For instance, ERK/MAPK signaling, mTOR signaling, JAK/STAT, or 14–3-3-mediated signaling were found to be significantly inhibited after 5.0 min SAG treatment but activated after treatment with vismodegib. We observed activation of 14–3-3-mediated signaling and Protein Kinase A signaling after 5.0 min of vismodegib treatment, consistent with a previous study showing that 14–3-3 epsilon binds to GLI after phosphorylation by Protein Kinase A, resulting in the inhibition of HH pathway activity [63].

The most significantly enriched pathways after 5.0 min treatment were Protein Kinase A signaling, Insulin Receptor signaling, as well as ERK/MAPK signaling (Fig. 3b). Interestingly, the majority of the phosphosites in the respective pathways was found to be downregulated after 5.0 min SAG treatment, whereas upregulation was predominantly observed after vismodegib treatment.

Protein Kinase A has been recognized to negatively modulate the hedgehog pathway and thus influence cell fate and proliferation [64]. In accordance with the literature we determined PKA signaling as activated after vismodegib treatment while inhibited after SAG treatment (Fig. 3). Intriguingly, this initial activation of PKA signaling after 5.0 min vismodegib treatment is not maintained after 15 min (Figure S-4A). The initial phosphorylation of PKA substrates after 5.0 min might induce downstream processes and appears to be balanced with time. PKA signaling was clearly inactivated after 15 min vismodegib treatment compared to 5.0 min as shown in Figure S-5. We confirmed activation and inactivation of PKA by vismodegib and SAG, respectively by Western blot analysis using activation-specific anti-phopho-PKA antibodies (see suppl. Figure S-6). Furthermore, Insulin Receptor Signaling is known to synergize with sonic HH in medulloblastoma formation [65] and was significantly enriched in our data set. Surprisingly, 5.0 min of vismodegib treatment activated Insulin Receptor signaling, while SAG treatment impeded the pathway, which may be explained by the partial agonistic effect reported for therapeutic SMO inhibitors [39]. However, SAG activates Insulin Receptor Signaling over time (Figure S-5) which was clearly induced after 15 min SAG treatment compared to 5.0 min treatment.

Additionally, we find ERK/MAPK signaling both enriched and activated after 5.0 min vismodegib treatment (Fig. 3). Extensive research has been conducted to decipher the interactions between sonic HH and ERK/MAPK signaling, however with contradictory results [40, 66, 67]. The regulation of HH signaling by ERK/MAPK signaling has been validated biochemically [68] and observed in multiple cancer entities such as non-melanoma and melanoma skin cancer [69–72], and gastric cancer [73], and hepatocellular carcinoma [74]. This has led to the suggestion to combine MEK or ERK1/2 inhibitors and HH pathway inhibitors to synergistically fight human cancers [75–78]. However, our data supports a negative crosstalk between HH and ERK/MAPK signaling since an activation of ERK/MAPK signaling was observed after vismodegib treatment. Of note, a negative interaction of HH and MEK/ERK signaling has also been reported by Götschel et al. [40] and by Neill et al. [79] for epidermal cells, suggesting context-dependent pathway interactions. Furthermore, we find increased activation of ERK/MAPK signaling after 15 min compared to 5.0 min SAG treatment (Figure S-5). Hence, SAG treatment induced ERK/MAPK signaling in a time-dependent manner indicating a slower kinetics compared to vismodegib treatment.

Research on the crosstalk of sonic HH signaling and the mTOR/S6K1 pathway revealed an activation of GLI1 in a SMO-independent manner indicating a positive crosstalk via non-canonical HH pathway activation [80]. In contrast, in our short-term pathway activation study, we find mTOR signaling to be activated after vismodegib but inhibited after SAG treatment, implying a negative crosstalk of mTOR signaling and the HH pathway.

Moreover, our data indicates that SAG and vismodegib treatment differentially influences JAK/STAT signaling (Fig. 3a). A positive crosstalk of sonic HH signaling with STAT3 was found in basal cell carcinoma [81, 82] and in human papillary thyroid cancer [83], but to our knowledge has not yet been reported for medulloblastoma. We find JAK/STAT signaling activated after 5.0 min of vismodegib treatment, while being inhibited after SAG treatment.

Interestingly, PTEN signaling was strongly inhibited after 5.0 min of vismodegib treatment. In contrast to our results, Hartmann et al. reported a link between PTEN loss and proliferation of medulloblastoma cells on genomic and epigenetic levels [84]. However, our data indicates that short-term vismodegib treatment can inhibit PTEN signaling in human medulloblastoma cells via phosphorylation cascades, demonstrating an additional level of crosstalk distinct from the previously reported. Likewise, Metcalfe et al. showed that allografts with PTEN deficient medulloblastoma do respond to vismodegib treatment, and therefore, still depend on sonic HH signaling [85]. Interestingly, SAG treatment influenced PTEN signaling in a time-dependent manner as shown in Figure S-5. We find decreased activation of PTEN signaling after 15 min compared to 5.0 min SAG treatment. This indicates initial activation of PTEN signaling by SAG which diminishes with time. Similarly, increased activation of PI3K/AKT signaling, the antagonist of PTEN signaling, is observed after 15 min compared to 5.0 min SAG treatment (Figure S-5). The crosstalk of PI3K/AKT/mTOR signaling and HH signaling has been reported frequently [67, 80]. Our data points to an initial induction of PTEN signaling by SAG, which is then balanced out by activation of PI3K/AKT signaling. This time-dependent activation of PI3K/AKT pathway after initial activation of PTEN signaling represents a potential mechanism of action of non-canonical HH pathway activation. These findings go along with the study of Chaturvedi et al. who combined PI3K-mTOR inhibition with vismodegib treatment, showing therapeutic effects in medulloblastoma in vitro and in vivo [86].

These time-dependent dynamics in pathway activation and inhibition explain that the differences between SAG and vismodegib treatment on pathway level found after 5.0 min were not observable after 15 min treatment (Figure S-4A). Vismodegib treatment might induce faster phosphorylation dynamics than SAG, meaning both drugs show different kinetics. However, we identified an overlap of 4960 phosphopeptides after both time points as shown in Figure S-7. In total, we found even more phosphopeptides after 15 min treatment compared to 5.0 min, which can be explained by enhanced signaling cascades downstream of initial phosphorylation events. These downstream signaling cascades result in similar pathway activation and phosphorylation patterns after 15 min both for SAG and vismodegib treatment as depicted in Figure S-4B.

### BRCA1 phosphorylation and IFT172 phosphorylation may be involved in HH pathway inhibition and ciliary assembly

**Fig. 4:**
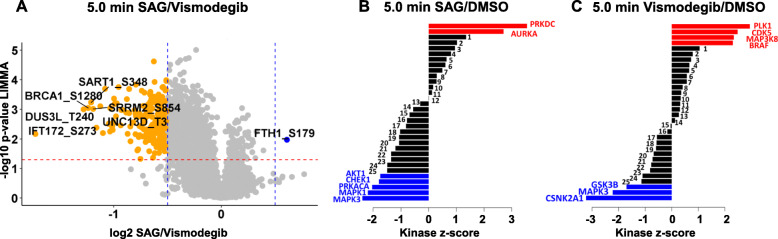
Volcano plot and kinase substrate enrichment analysis for phosphopeptides identified after 5.0 min treatment. **a** Distribution and significantly regulated phosphopeptides between SAG and vismodegib treatment. Ratios were determined by division of the ratio SAG/DMSO by the ratio vismodegib/DMSO: SAG/vismodegib. LIMMA statistical testing was applied to determine *p*-values and statistically significant regulated phosphopeptides. Orange and blue dots, significantly down- or upregulated phosphopeptides, respectively (threshold of *p* < 0.05.0); log2-fold-change cutoff was set to 0.5 and – 0.5 (**b**) and (**c**) Kinase Substrate Enrichment Analysis. Proteome normalized ratios of SAG/DMSO (**b**) and Vismo/DMSO (**c**) were taken to infer the kinase activation score with a *p*-value cutoff of 0.05. Significantly activated kinases are presented in red, significantly attenuated kinases in blue. A table with enriched kinases with corresponding numbers is attached in the supplementary material

LIMMA significance testing was applied on the proteome-normalized intensity values of the three biological replicates of respective treatments. *P*-values were determined to evaluate the statistical significance of up- and downregulated phosphopeptides after 5.0 and 15 min treatment. Statistical significance was evaluated for each treatment relative to DMSO as control treatment and between both treatments SAG and vismodegib. Whereas only one phosphopeptide was found to be significantly upregulated after 5.0 min SAG treatment compared to DMSO as control (Figure S-8A), 18 phosphopeptides were significantly upregulated after vismodegib treatment (Figure S-8B). Even 216 phosphopeptides were significantly regulated between both treatments after 5.0 min with a significance threshold of *p* < 0.05. The corresponding volcano plot is shown in Fig. 4a. Here, the ratio of SAG/vismodegib reflects the ratio of SAG/DMSO divided by vismodegib/DMSO, hence negative ratios represent an upregulation after vismodegib treatment compared to SAG treatment.

Interestingly, phosphorylation of Breast cancer type 1 susceptibility protein (BRCA1) at serine 1280 was significantly upregulated after HH pathway inhibition (Fig. 4a). BRCA1 plays important roles in the repair of DNA damage. ATM-dependent phosphorylation of BRCA1 in response to DNA damage has been found by Cortez et al. [87]. ATM-related kinase ATR has been described to phosphorylate BRCA1 at serine 1280, promoting its delocalization into the nucleus [88, 89]. Furthermore, phosphorylation of BRCA1 by AURKA is known to inhibit BRCA1 activity [90]. In accordance with these findings, kinase substrate enrichment revealed AURKA as significantly activated after 5.0 min SAG treatment, i.e. HH pathway induction (Fig. 4b). Furthermore, AURKA was the most significantly activated kinase after kinase substrate enrichment of 5.0 min SAG compared to vismodegib treatment (Figure S-9). AURKA has been implicated in regulating the proliferation and growth of human glioma cells [91], and in the (dis) assembly of the primary cilium. In line with its role in tumor cell proliferation, inhibitors of AURKA have successfully been applied in the treatment of tumor-propagating cells in medulloblastoma mouse models [92].

Furthermore, Intraflagellar transport protein 172 homolog (IFT172) was significantly higher phosphorylated at serine 273 after vismodegib treatment compared to SAG treatment. IFT172 is part of Intraflagellar transport complex B (IFT B) and important for ciliary assembly and essential for brain development [93]. Our point to a possible role of Ser273 phosphorylation of IFT172 in the regulation of ciliary transport of effector proteins involved in HH signaling. It will be interesting to address in future studies whether this phosphorylation event influences the regulation and coordination of HH signaling in the primary cilium.

### Polo-like-kinase 1 activation and casein kinase 2 A1 inhibition by vismodegib treatment

Kinase substrate enrichment analysis of phosphopeptides identified after 5.0 min treatment revealed activation of Polo-like kinase 1 (PLK1) after vismodegib treatment along with CDK5, MAP 3 K8 and BRAF (Fig. 4c). Like AURKA, Polo-like Kinase-1 (PLK1) has been implicated in the control of cilia formation. It will therefore be important to address the role of these kinases in the regulation of HH signaling at the level of the primary cilium [94, 95] and in the context of therapeutic interventions using SMO modifiers. PLK1 is best known for its positive function in G2/M phase progression [96] and therefore has been intensely investigated as an attractive drug target for cancer therapy [97]. In accordance with our data, Zhang et al. found PLK1 as a negative regulator of Hh signaling by phosphorylation of GLI1 [98]. In contrast to our findings, Polo-like kinase-1 inhibition by small molecules was found to be beneficial in the treatment of HH dependent medulloblastoma cell lines such as DAOY cells [99].

Furthermore, Casein Kinase 2A1 (CSNK2A1) was predicted to be significantly downregulated after 5.0 min vismodegib treatment (Fig. 4c). Casein Kinase 2 is known as an important positive regulator of Hh signaling in Drosophila [100] and lung cancer cell lines [101]. In accordance with our data, Casein Kinase 2 was recently identified as a major regulator of medulloblastoma [102] and described as a key driver of HH signaling in murine granule neuron precursors [102].

### Significant changes in phosphorylation of HH pathway components are likely to drive ciliary assembly after 5.0 min and signal transduction in the primary cilium after 15 min

**Fig. 5:**
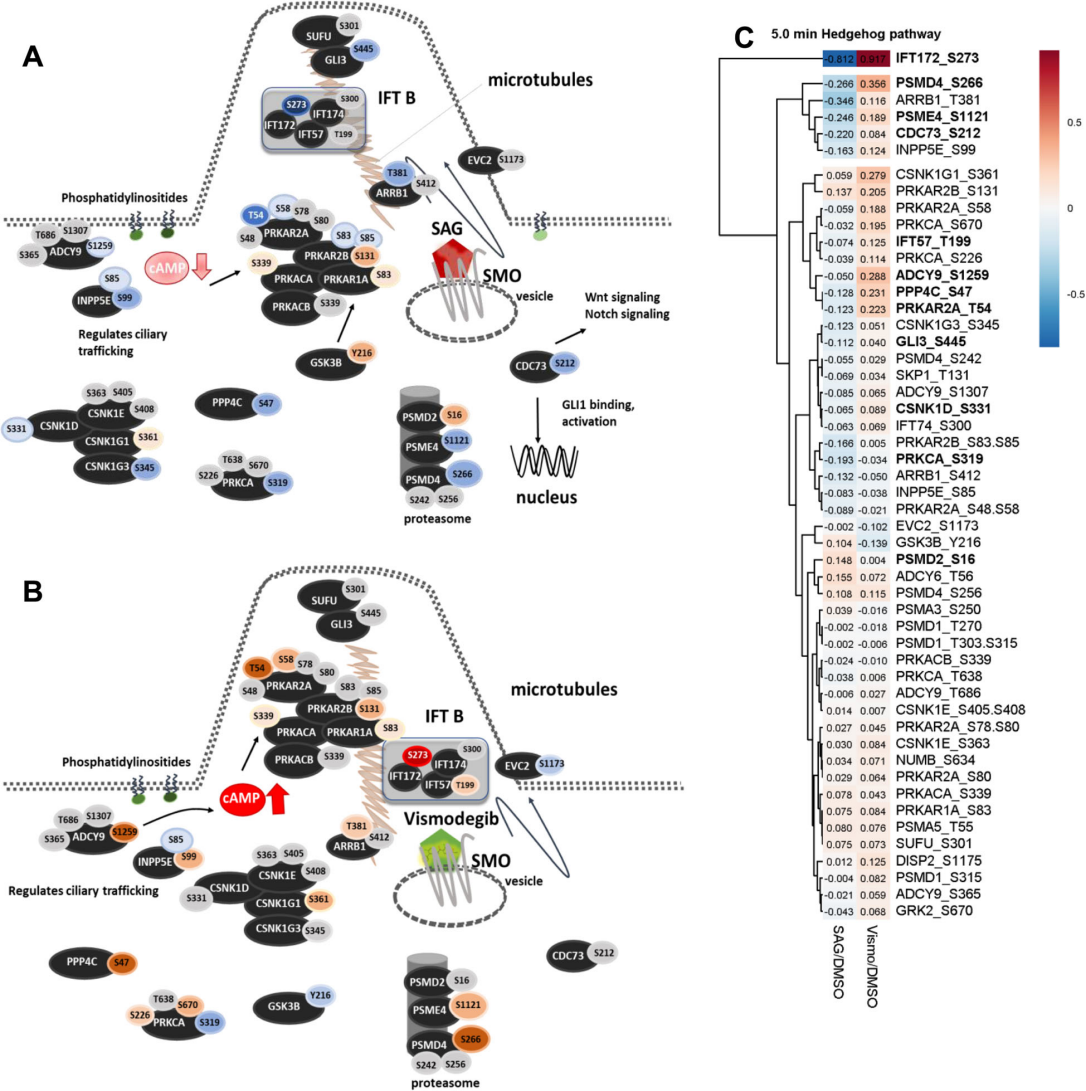
Phosphorylation of HH pathway components after 5.0 min treatment. **a** SAG binding to Smoothened induces ciliary trafficking after 5.0 min stimulation. **b** Vismodegib treatment induces phosphorylation and increases cAMP levels after 5.0 min treatment. **c** Heatmap of regulated and identified phosphosites after 5.0 min SAG and vismodegib treatment. Blue and red ellipses represent phosphosites down- or upregulated compared to control. All quantified phosphopeptides of phosphoproteins after 5.0 min treatment assigned to sonic hedgehog signaling were considered for the heatmap without applying any cut-off value. SAG/DMSO represents the ratio determined by the mean of the proteome-normalized intensities detected after SAG treatment (*N* = 3) divided by the mean of proteome-normalized intensities after DMSO (*N* = 3). Vismo/DMSO represents the ratio determined by the mean of proteome-normalized intensities detected after vismodegib treatment (*N* = 3) divided by the mean of proteome-normalized intensities after DMSO (*N* = 3). Significantly regulated phosphopeptides between SAG and vismodegib treatment are highlighted in bold. Significantly regulated phosphopeptides between SAG and vismodegib treatment are highlighted in bold

**Fig. 6:**
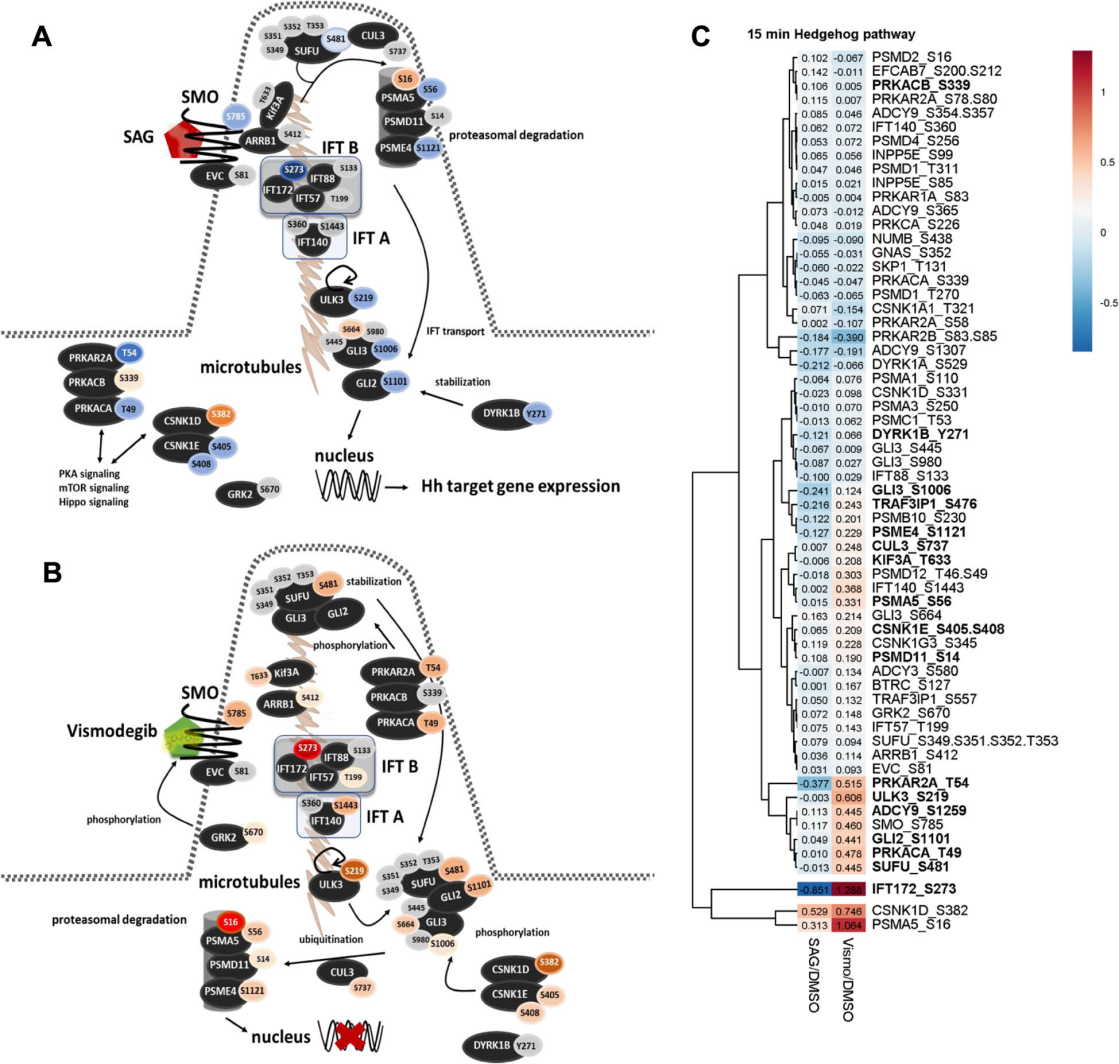
Phosphorylation of HH pathway components after 15 min treatment. **a** SAG impairs GLI phosphorylation after 15 min stimulation. **b** Vismodegib binding induces delocalization of Smoothened in the primary cilium. **c** Heatmap of regulated and identified phosphosites after 15 min SAG and vismodegib treatment. Blue and red ellipses, phosphosites down- or upregulated compared to control. All quantified phosphopeptides of phosphoproteins after 15 min treatment assigned to sonic hedgehog signaling search were considered for the heatmap without applying any cutoff value. SAG/DMSO represents the ratio determined by the mean of the proteome-normalized intensities detected after SAG treatment (*N* = 3) divided by the mean of proteome-normalized intensities after DMSO (*N* = 3). Vismo/DMSO represents the ratio determined by the mean of proteome-normalized intensities detected after vismodegib treatment (*N* = 3) divided by the mean of proteome-normalized intensities after DMSO (*N* = 3). Significantly regulated phosphopeptides between SAG and vismodegib treatment are highlighted in bold

We filtered the phosphoproteome of each time point for proteins, which are involved in or linked to HH signaling. Interestingly, the quantitative changes in the phosphoproteome of HH pathway components were rather low. However, various differences in the phosphorylation pattern of particular components of the Hedgehog pathway were observable. Pathway maps relying on identified phosphopeptides and the corresponding heatmap of HH pathway phosphoproteins are presented in Fig. 5 for 5.0 min and Fig. 6 for 15 min treatment.

In total, we identified 51 phosphopeptides involved in HH signaling after 5.0 min treatment, of which 12 phosphopeptides were significantly different between SAG and vismodegib treatment (corresponding proteins highlighted in bold in Fig. 5c). Various phosphosites of Protein Kinase A subunits, Casein Kinase 1 subunits, and also Protein Kinase C were identified after 5.0 min treatment and indicated in Fig. 5a and b. In particular threonine 54 of cAMP-dependent Protein Kinase type II-alpha regulatory subunit (PRKAR2A) was significantly downregulated after SAG and highly upregulated after vismodegib treatment. GSK3β was found to be phosphorylated and thus activated at tyrosine 216 after SAG treatment. Both kinases play central regulatory roles in sonic HH signaling [27, 32, 103].

Moreover, we found phosphorylation of the catalytic subunit of the phosphatase PPP4C, which is involved in activation and deactivation of PP4 for SMO dephosphorylation after 5.0 min treatment. Ser47 phosphorylation of PPP4C was significantly downregulated after SAG treatment and upregulated after vismodegib treatment. In accordance with our observation, Jia et al. reported a negative regulatory function of PP4 in SMO phosphorylation and HH pathway transduction [36]. However, PP4 controls microtubule organization and is implicated in various cellular processes [104]. Hence, the differences in PP4 phosphorylation could also reflect its functional role in microtubular coordination for signal transduction, e.g. in the primary cilium.

Interestingly, proteasomal subunits such as PSMD4 and PSME4 were differentially phosphorylated after both treatments, suggesting that post-translational modification of the proteasome machinery is an early response to SMO modulation. Moreover, various Adenylylcyclases and 72 kDa Inositol polyphosphate 5.0-phosphatase INPP5E were differentially phosphorylated after 5.0 min SAG and vismodegib treatment. Ciliary phosphoinositides control ciliary trafficking and thus contribute to the regulation of HH signaling [105]. INPP5E in particular regulates the level of inhibitors of Hh signaling in the primary cilium [106]. Moore et al. showed that cilia have a high cAMP level which is controlled by PKA and phosphoinositide dependent signaling [107]. In accordance with these findings we observe inactivation of Adenylylcyclases, INPP5E and PKA via dephosphorylation in response to activation of the HH pathway by 5.0 min SAG treatment. This observation is in line with the current model of reduced PKA activity in response to SMO activation and thus provides a basis for future studies to unravel in detail the elusive molecular link between SMO, PKA, SUFU and GLI.

Intriguingly and consistent with the effect of SAG, vismodegib treatment induced rapid phosphorylation of PKA subunits and the components of phosphoinositol signaling, indicating higher levels of cAMP and PKA activity in the cilium, which is required for the formation of GLI repressor forms. Our data also point to a role of IFT172 in the ciliary regulation and regulation of HH signaling. It represents a part of the IFT B complex and its phosphorylation was found to be highly downregulated after SAG and upregulated after vismodegib treatment at serine 273. Intraflagellar transport is crucial for ciliary trafficking and assembly, and thus for the control of HH signaling [108]. Corresponding to our findings, it has already been shown that disruption of IFT172 led to a complete lack of primary cilia [109]. Protein phosphorylation was reported to be important for flagellar disassembly by Pan et al. [110] but the importance of serine 273 phosphorylation at IFT172 was not demonstrated before. This phosphosite remains significantly regulated also after 15 min treatment.

Hence, we propose the following model of the HH pathway for the 5.0 min time point, visualized in Fig. 5a and b. SAG treatment induces dephosphorylation of central phosphoproteins of the Intraflagellar Transport complex (IFT) B and beta-Arrestin, which leads to the transport of Smoothened into the cilium. Furthermore, the ciliary cAMP level is reduced by dephosphorylation of ADCY9 and INPP5E resulting in inhibition of ciliary Protein Kinase A and other cytosolic kinases such as Casein kinases and Protein Kinase C. Protein Kinase A delocalizes into the cytoplasm and interacts with GSK3β which is activated by phosphorylation at Tyrosine 216. Dephosphorylation of CDC73 further activates sonic HH signaling by binding to GLI1 on gene level. In contrast, vismodegib binding to Smoothened causes phosphorylation of intraflagellar transport proteins in particular IFT172 (serine 273). ADCY9 and INPP5E are phosphorylated resulting in increased cAMP levels and activation of ciliary Protein Kinase A and other cytosolic kinases such as Casein kinases and Protein Kinase C. GSK3β is inhibited by dephosphorylation at tyrosine 216.

We could identify additional phosphopeptides with a possible role in HH signaling after 15 min treatment (Fig. 6). Out of 61 phosphopeptides, 17 were significantly regulated between both treatments. Vismodegib treatment induced phosphorylation of central HH pathway components such as SMO, SUFU, GLI2 and GLI3. GLI phosphorylation is likely to be a consequence of increased PKA activity (see above), which is known to promote the proteasomal processing and degradation, respectively [103]. Furthermore, SUFU phosphorylation stabilizes the inhibitory SUFU-GLI complex [32]. In contrast, SAG treatment induced dephosphorylation at these phosphosites possibly promoting proteasomal degradation of SUFU and activation of the GLI transcription factors.

Moreover, Serine/threonine-protein Kinase ULK3 (ULK3) was found to be significantly higher phosphorylated after 15 min of vismodegib treatment compared to SAG treatment (Fig. 6). A dual function of ULK3 in the regulation of the HH pathway was already reported 2010 by Maloverjan et al. [111, 112] They showed that ULK3 both interacts with SUFU and GLI2. Our data shows that 15 min vismodegib treatment induces ULK3 phosphorylation at serine 219 while SAG treatment reduces phosphorylation at this phosphosite compared to control treatment. The phosphorylation of ULK3 at serine 219 was also significantly upregulated after vismodegib treatment compared to DMSO treatment as visualized in the volcano plot in Figure S-10 B. Interestingly, Kinesin-like protein Kif3A phosphorylation at Thr633 was significantly increased after 15 min vismodegib treatment. Kif3A is essential for HH pathway activation [113] and required for ciliary trafficking of Smoothened [114]. Furthermore, the significant upregulation at Ser273 of IFT172 observed after 5.0 min vismodegib treatment persists after 15 min. An increase in the phosphorylation from 5.0 to 15 min can be observed only after vismodegib treatment. In contrast, IFT172 expression levels do not change over time and phosphorylation does not change after SAG treatment as shown in Figure S-11. Our data thus supports a model where phosphorylation serves as a regulatory mechanism to control ciliary trafficking of central components of the HH pathway, possibly via Kif3A and its function in the primary cilium. Phosphorylation events are also likely to impact on the stability of the SUFU-GLI complex, which may involve differential phosphorylation of ULK3 in response to SMO modulation.

In sum, we come up with the following effects on the phosphorylation dynamics within the HH pathway after 15 min SAG and vismodegib treatment as depicted in Fig. 6a and b. Upon SAG treatment, ARRB1 and Kif3A bind to dephosphorylated Smoothened and facilitate the delocalization into the primary cilium. SUFU gets dephosphorylated at Ser481 and Cul3 facilitates its proteasomal degradation. GLI2 and GLI3 are released from the SUFU complex and mostly dephosphorylated. SUFU is ubiquitinated and subjected to proteasomal degradation. GLI2 and GLI3 are transported by intraflagellar transport proteins of the IFT A and B complex. Dephosphorylation of DYRK1B (Y271) stabilizes GLI2 in its activator form, leading to Hh target gene expression. Protein Kinase A is mostly inactivated by dephosphorylation. On the other hand, Smoothened gets phosphorylated after 15 min vismodegib treatment by GRK2 at Ser785. SUFU is further phosphorylated at Ser481, which stabilizes the complex to GLI2 and GLI3. IFT B and IFT A proteins get phosphorylated and facilitate the transport of the SUFU-Gli complex into the cytosol. Ciliary protein Kinase A and cytoplasmic Casein kinases contribute to phosphorylation of GLI2 and GLI3. ULK3 is phosphorylated and interacts with SUFU promoting the CUL3 driven ubiquitination of GLI2 and GLI3 and their subsequent proteasomal degradation.

## Conclusions

Monitoring of close to 10,000 phosphopeptides of human medulloblastoma upon short-term stimulation with SMO activators and inhibitors, respectively, allowed us to investigate the complex early phosphosignaling during HH pathway regulation. Changes in the phosphoproteome in response to activation or inhibition of the HH pathway are already observed after 5.0 min of treatment and mainly affect signaling pathways involved in cellular growth and proliferation. Of note, rapid changes in phosphorylation patterns of HH pathway components mainly affect components of the primary cilium as well as signaling via the SUFU-GLI axis. Intraflagellar transport proteins such as IFT172 were differentially phosphorylated indicating a crucial role of phosphorylation in the regulation of the intraflagellar transport machinery and hence the transport of HH pathway components in the primary cilium. Furthermore, phosphorylation influences Protein Kinase A signaling besides PI3K/AKT/mTOR and PTEN signaling as we find these pathways differentially regulated by phosphorylation dynamics upon short-term activation and inhibition of the HH pathway.

Our data suggests that several kinases play important roles in the regulation of short-term activation or inhibition of HH signaling. Aurora Kinase A may be involved in the activation of HH signaling upon SAG treatment. El-Sheikh et al. already showed that inhibition of Aurora Kinase A enhances the chemosensitivity of medulloblastoma [115], and targeting Aurora Kinase A in combination with HH pathway inhibitors was reported as novel therapeutic strategy in the treatment of human medulloblastoma [92]. However, inhibition of HH signaling simulated by vismodegib treatment was dominated by Polo-like Kinase-1 (PLK1). PLK1 was only recently shown to negatively regulate sonic HH signaling [98], which goes along with our findings. Furthermore, inhibition of Casein Kinase 2A1 after short-term vismodegib treatment underlines the discovery of Casein Kinase 2 as a key driver of hedgehog signaling by Purzner et al. [26]. However, our data highlights the involvement of subunit alpha in this functional role.

Elucidation of these deep phosphorylation cascades in HH signaling paves the way to a profound understanding of the basis of the immediate events of HH pathway activation important for the development of targeted therapies of sonic HH-type medulloblastoma patients. The new insights into changes in the phosphorylation landscape upon vismodegib treatment clears up its mechanism of action and will be beneficial for the prevention of adverse effects and therapeutic resistance.

## Endnote

^1^The content is solely the responsibility of the authors and does not necessarily represent the official views of the National Institutes of Health.

## Supplementary information

**Additional file 1: Table S-1.** Eluent B (B) gradient range and gradient duration for the different analyzed fractions. **Table S-2.** Applied R-packages. Responsiveness of DAOY cells to SAG and vismodegib treatment. RNA isolation and quantitative PCR (qPCR). **Table S-3.** Primer Sequences used for qPCR. **Figure S-1.** Hedgehog target gene expression in SAG and vismodegib treated DAOY cells. **Figure S-2.** Comparable Hedgehog pathway induction by natural and synthetic HH pathway activators. **Figure S-3.** Distribution of protein groups identified after 5.0 min treatment (A) and 15 min treatment (B). **Figure S-4.** Ingenuity pathway analysis of all phosphopeptides identified after 15 min using a phosphorylation analysis. **Figure S-5.** Time dependent Ingenuity pathway analysis of cancer associated pathways. **Figure S-6.** Western blot validation of PKA activity modulation in response to SMO agonist and antagonists. **Table S-4.** Antibodies used for Western blot analysis. **Figure S-7.** Overlap of quantified phosphopeptides after 5.0 and 15 min. **Figure S-8.** Volcano plots of phosphopeptides identified after 5.0 min treatment. **Figure S-9.** Kinase substrate enrichment analysis (KSEA) was performed for phosphopeptides identified after 5.0 min treatment for the ratio SAG/Vismo using the online platform KSEA App (https://casecpb.shinyapps.io/ksea/). **Figure S-10.** Volcano plots and kinase set enrichment analysis for phosphopeptides identified after 15 min treatment. **Figure S-11.** IFT172 phosphorylation and expression after 5.0 and 15 min.

## Data Availability

The mass spectrometry proteomics data have been deposited to the ProteomeXchange Consortium via the PRIDE partner repository with the dataset identifier PXD013408. Reviewer account details: Username: reviewer62442@ebi.ac.uk Password: NPewuMva Further data has been attached: Proteomics data: Protein lists with all quantified proteins and corresponding ratios (5min_pH8_Ratios.csv; 15min_pH8_Ratios.csv) Phosphoproteomics data: Phosphopeptidelists with all confident phosphopeptides with normalized raw intensity values (FLR < 0.05.0), ratios SAG_DMSO, Vismo_DMSO and EGF_DMSO and *p*-values according to LIMMA statistical testing for SAG vs DMSO (SD), Vismo vs DMSO (VD) and SAG vs Vismo (SV): 5min_HILIC_final.csv, 15min_HILIC_final.csv Supplementary figures (pdf) List of kinases of Fig. 4 (KSEA_kinases_Figure4.xlsx) KNIME workflows applied for proteomics and phosphoproteomics raw data analysis and R scripts applied for further data filtering and visualization can be accessed here: Phosphoproteomics Workflows: 5 min: http://www.myexperiment.org/workflows/5113.html 15 min: http://www.myexperiment.org/workflows/5114.html Proteomics Workflows: 5 min: http://www.myexperiment.org/workflows/5115.html 15 min: http://www.myexperiment.org/workflows/5116.html

## Abbreviations

ADCY9: Adenylate cyclase type 9
ARRB1: Beta-arrestin-1
ATM: Serine protein kinase ATM
ATR: ATM-related kinase
AURKA: Aurora kinase A
BRAF: Serine/threonine-protein kinase B-raf
BRCA 1: Breast cancer type 1 susceptibility protein
cAMP: Cyclic adenosine monophosphate
CDC73: Parafibromin
CDK5: Cyclin-dependent-like kinase 5
CSNK2A1: Casein Kinase 2A1
Cul3: Cullin-3
DTT: Dithiothreitol
DYRK1B: Dual specificity tyrosine-phosphorylation-regulated kinase 1B
EGF: Epidermal Growth Factor
ERK: Extracellular Signal regulated kinase
GLI: Glioma-associated oncogene
GRK2: Beta-adrenergic receptor kinase 1
GSK3β: Glycogen synthase kinase 3β
HH: Hedgehog
HILIC: Hydrophilic interaction liquid chromatography
HPLC: High performance liquid chromatography
IFT B: Intraflagellar transport complex B
IFT: Intraflagellar Transport
IFT172: Intraflagellar transport protein 172 homolog
INPP5E: 72 kDa inositol Polyphosphate 5-phosphatase
IPA: Ingenuity Pathway Analysis
JAK: Januskinase
KIF 3A: Kinesin-like protein KIF3A
Kif7: Kinesin-like protein KIF7
LIMMA: Linear Models for Microarray
MAP 3K8: Mitogen-activated protein kinase kinase kinase 8
MAPK: Mitogen-activated protein kinase
MS: Mass spectrometry
mTOR: Mechanistic Target of Rapamycin
PI3K: Phosphatidylinositol 3-kinases
PKA: Protein Kinase A
PLK1: Polo-like kinase 1
PLK1: Polo-like kinase-1
PP2A: Protein phosphatase 2
PP4: Protein phosphatase 4
PPP4C: Serine/threonine-protein phosphatase 4 catalytic subunit
PRKAR2A: cAMP-dependent Protein Kinase type II-alpha regulatory subunit
PSMD4: 26S proteasome non-ATPase regulatory subunit 4
PSME4: Proteasome activator complex subunit 4
PTCH 1: Patched 1
PTEN: Phosphatidylinositol 3,4,5-trisphosphate 3-phosphatase and dual-specificity protein phosphatase
PTM: Post-translational Modification
Ras: Rat Sarcoma
S6K1: Ribosomal protein S6 kinase beta-1
SAG: Smoothened Agonist
SMO: Smoothened
STAT: Signal Transducers and Activators of Transcription
SUFU: Suppressor of Fused
TEAB: Triethylammonium bicarbonate
TFA: Trifluoroacetic acid
ULK3: Serine/threonine-protein kinase ULK3
Vismo: Vismodegib
