# Supplementary material for: Phosphoproteomics of short-term hedgehog signaling in human medulloblastoma cells
Source: Cell Commun Signal. 2020 Jun 23;18:99. doi: 10.1186/s12964-020-00591-0 (PMC7310537; doi:10.1186/s12964-020-00591-0)
Supplement: Supplementary file 2 — Additional file 1: Table S-1. Eluent B (B) gradient range and gradient duration for the different analyzed fractions. Table S-2. Applied R-packages. Responsiveness of DAOY cells to SAG and vismodegib treatment. RNA isolation and quantitative PCR (qPCR). Table S-3. Primer Sequences used for qPCR. Figure S-1. Hedgehog target gene expression in SAG and vismodegib treated DAOY cells. Figure S-2. Comparable Hedgehog pathway induction by natural and synthetic HH pathway activators. Figure S-3. Distribution of protein groups identified after 5.0 min treatment (A) and 15 min treatment (B). Figure S-4. Ingenuity pathway analysis of all phosphopeptides identified after 15 min using a phosphorylation analysis. Figure S-5. Time dependent Ingenuity pathway analysis of cancer associated pathways. Figure S-6. Western blot validation of PKA activity modulation in response to SMO agonist and antagonists. Table S-4. Antibodies used for Western blot analysis. Figure S-7. Overlap of quantified phosphopeptides after 5.0 and 15 min. Figure S-8. Volcano plots of phosphopeptides identified after 5.0 min treatment. Figure S-9. Kinase substrate enrichment analysis (KSEA) was performed for phosphopeptides identified after 5.0 min treatment for the ratio SAG/Vismo using the online platform KSEA App (https://casecpb.shinyapps.io/ksea/). Figure S-10. Volcano plots and kinase set enrichment analysis for phosphopeptides identified after 15 min treatment. Figure S-11. IFT172 phosphorylation and expression after 5.0 and 15 min. [file 12964_2020_591_MOESM2_ESM.docx]

Scheidt et al. supplementary material

Phosphoproteomics of short-term Hedgehog signaling in human medulloblastoma cells

Tamara Scheidt^1^, Oliver Alka^2^, Humberto Gonczarowska-Jorge^3,a^, Wolfgang Gruber^1,b^, Florian Rathje^1^, Margherita Dell’Aica^3^, Marc Rurik^2^, Oliver Kohlbacher^2,4,5,6^, René P. Zahedi^3,7^, Fritz Aberger^1^, Christian G. Huber^1*^

^1^Department of Biosciences, Cancer Cluster Salzburg, University of Salzburg, Salzburg, Austria

^2^Institute for Bioinformatics and Medical Informatics, University of Tübingen, Sand 14, 72076 Tübingen, Germany

^3^Leibniz-Institute of Analytical Sciences- ISAS - e.V., Dortmund, Germany

^4^Biomolecular Interactions, Max Planck Institute for Developmental Biology, Max-Planck-Ring 5, 72076 Tübingen, Germany

^5^Institute for Translational Bioinformatics, University Hospital Tübingen, Hoppe-Seyler-Str. 9, 72076 Tübingen, Germany

^6^Quantitative Biology Center, University of Tübingen, Auf der Morgenstelle 10, 72076 Tübingen, Germany

^7^Gerald Bronfman Department of Oncology, Jewish General Hospital, McGill University, Montreal, Canada; Segal Cancer Proteomics Centre, Lady Davis Institute, Jewish General Hospital, McGill University, Montreal, Canada.

^a^present address: CAPES Foundation, Ministry of Education of Brazil, Brasília, DF 70040-020, Brazil.

^a^present address: EVER Valinject GmbH, 4866 Unterach am Attersee, Austria

Running title: phosphosignaling in the Sonic Hedgehog pathway

Table of contents

**Table S-1.** Eluent B (B) gradient range and gradient duration for the different analyzed fractions. p. 4

**Table S-2.** Applied R-packages. p. 5-6

Responsiveness of DAOY cells to SAG and vismodegib treatment. p. 7

RNA isolation and quantitative PCR (qPCR). p. 7

**Table S-3.** Primer Sequences used for qPCR. p.7

**Figure S-1.** Hedgehog target gene expression in SAG and vismodegib treated DAOY cells

p. 8

**Figure S-2.** Comparable Hedgehog pathway induction by natural and synthetic HH pathway activators. p. 9

**Figure S-3.** Distribution of protein groups identified after 5.0 min treatment (A) and 15 min treatment (B). p. 10

**Figure S-4.** Ingenuity pathway analysis of all phosphopeptides identified after 15 minutes using a phosphorylation analysis. p. 11-12

**Figure S-5.** Time dependent Ingenuity pathway analysis of cancer associated pathways p.13

**Figure S-6.**  Western blot validation of PKA activity modulation in response to SMO agonist and antagonists p. 14

**Table S-4.** Antibodies used for Western blot analysis. p. 14

**Figure S-7.** Overlap of quantified phosphopeptides after 5.0 and 15 min. p.15

**Figure S-8.** Volcano plots of phosphopeptides identified after 5.0 minutes treatment. p.16

**Figure S-9.** Kinase substrate enrichment analysis (KSEA) was performed for phosphopeptides identified after 5.0.0 minutes treatment for the ratio SAG/Vismo using the online platform KSEA App (casecpb.shinyapps.io/ksea/). p.17

**Figure S-10.** Volcano plots and kinase set enrichment analysis for phosphopeptides identified after 15 minutes treatment. p.18-19

**Figure S-11.** IFT172 phosphorylation and expression after 5.0 and 15 min. p.20

References p.21

**Table S-1.** Eluent B (B) gradient range and gradient duration for the different analyzed fractions.

| Experiment | Main column gradient (B) | Gradient duration (min) |
| --- | --- | --- |
| High pH reversed phase fractionation | 3-35.0% | 90 |
| HILIC fractions 1, 12 | 3-35.0% | 40 |
| HILIC fractions 2, 3, 9 | 3-35.0% | 60 |
| HILIC fractions 4, 5.0, 6, 7, 8 | 5.0-42% | 120 |

**Table S-2.** Applied R-packages

| R-package | Version | Citation |
| --- | --- | --- |
| tidyverse | 1.2.1 | Hadley Wickham (2017). tidyverse: Easily Install and Load the 'Tidyverse'. R package version 1.2.1. https://CRAN.R-project.org/package=tidyverse |
| dplyr | 0.7.6 | Hadley Wickham, Romain François, Lionel Henry and Kirill Müller (2018). dplyr: A Grammar of Data Manipulation. R package version 0.7.6. https://CRAN.R-project.org/package=dplyr |
| org.Hs.eg.db | 3.6.0 | Marc Carlson (2018). org.Hs.eg.db: Genome wide annotation for Human. R package version 3.6.0. |
| limma | 3.36.5.0 | Ritchie, M.E., Phipson, B., Wu, D., Hu, Y., Law, C.W., Shi, W., and Smyth, G.K. (2015). limma powers differential expression analyses for RNA-sequencing and microarray studies. Nucleic Acids Research 43(7), e47. |
| ggrepel | 0.8.0 | Kamil Slowikowski (2018). ggrepel: Automatically Position Non-Overlapping Text Labels with 'ggplot2'. R package version 0.8.0. https://CRAN.R-project.org/package=ggrepel |
| lattice | 0.20-35.0 | Sarkar, Deepayan (2008) Lattice: Multivariate Data Visualization with R. Springer, New York. ISBN 978-0-387-75.0968-5.0 |
| RColorBrewer | 1.1-2 | Erich Neuwirth (2014). RColorBrewer: ColorBrewer Palettes. R package version 1.1-2. https://CRAN.R-project.org/package=RColorBrewer |
| MASS | 7.3-5.00 | Venables, W. N. & Ripley, B. D. (2002) Modern Applied Statistics with S. Fourth Edition. Springer, New York. ISBN 0-387-95.045.07-0 |
| pheatmap | 1.0.10 | Raivo Kolde (2018). pheatmap: Pretty Heatmaps. R package version 1.0.10. https://CRAN.R-project.org/package=pheatmap |
| shiny | 1.3.2 | Winston Chang, Joe Cheng, JJ Allaire, Yihui Xie and Jonathan McPherson (2019). shiny: Web Application Framework for R. R package version 1.3.2.  https://CRAN.R-project.org/package=shiny |

**Responsiveness of DAOY cells to SAG and vismodegib treatment**

**Quantitative real-time PCR (qPCR).**

RNA isolation and qPCR analysis of HH/GLI target genes was done as described previously (Sternberg et al., 2018). qPCR was done on a Rotor‐Gene Q (Qiagen) using GoTaq 2× qPCR Mastermix (Promega). Primers for GLI1, PTCH and HHIP are listed in **Table S-3**.

| **Table S-3** | **Primer Sequences used for qPCR** | |
| --- | --- | --- |
| Gene name | forward sequence (5´-3´) | reverse sequence (5´-3´) |
| GLI1 | **GCCGTGCTAAAGCTCCAGTGAACACA** | **TCCCACTTTGAGAGGCCCATAGCAAG** |
| HHIP | **AGGCTGGGAGGGGGACTTCTGC** | **GCCCTGGTCACTCTGCGGATGTT** |
| PTCH | **TCCTCGTGTGCGCTGTCTTCCTTC** | **CGTCAGAAAGGCCAAAGCAACGTGA** |

**Figure S-1. Hedgehog target gene expression in SAG and vismodegib treated DAOY cells.** Quantitative real-time PCR analysis of Hedgehog target gene expression. DAOY cells were treated with 0.005% DMSO solvent (DMSO), 100 nM SAG (SAG) and 500 nM vismodegib (vismodegib) for 48 hours . Vismodegib was added 1 hour prior to SAG treatment . The HH/GLI target genes GLI1, PTCH and HHIP served as read-out for pathway activity. Data are represented by the mean of two independent replicate experiments. The error bars refer to the standard deviation. Relative mRNA levels are represented as 2^-dCt^ levels. d: delta; Ct: threshold cycle;


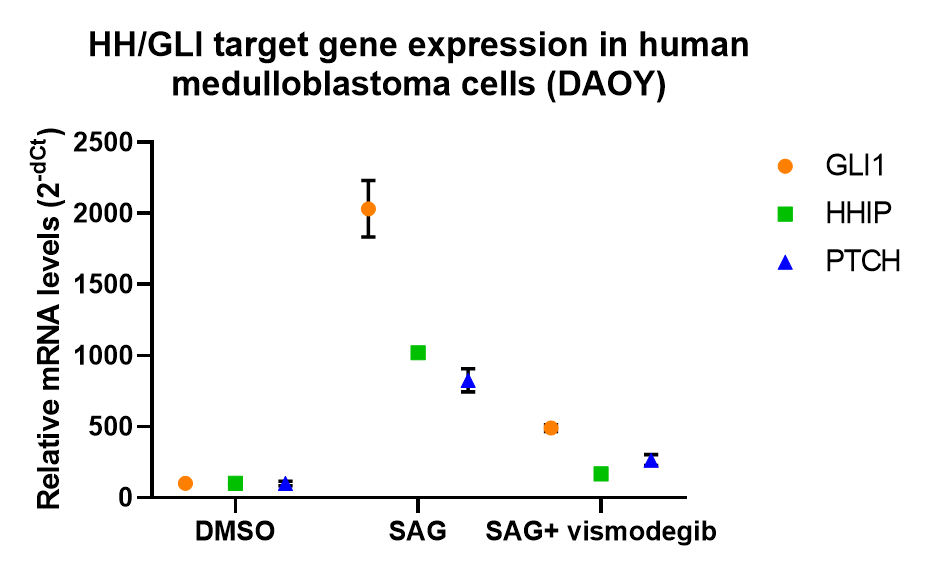


**Figure S-2. Comparable Hedgehog pathway induction by natural and synthetic HH pathway activators.** To compare the potency of natural versus synthetic HH pathway inducers, DAOY cells were treated after 2d of confluence in starving media (MEM, 0.5% FBS) with recombinant biologically active Sonic hedgehog protein (rShh, R&D Systems) or with the synthetic pathway activator SAG alone or in combination with the Smoothened antagonist vismodegib. After treatment for the time indicated, RNA samples were subject to qPCR analysis to determine the level and kinetics of HH target gene activation (i.e. activation of GLI1 (A) and HHIP (B) mRNA expression). Each treatment was done for 1 h, 3 h, 6 h, 12 h and 24 h. qPCR analysis was performed as described previously (Sternberg et al., 2018).


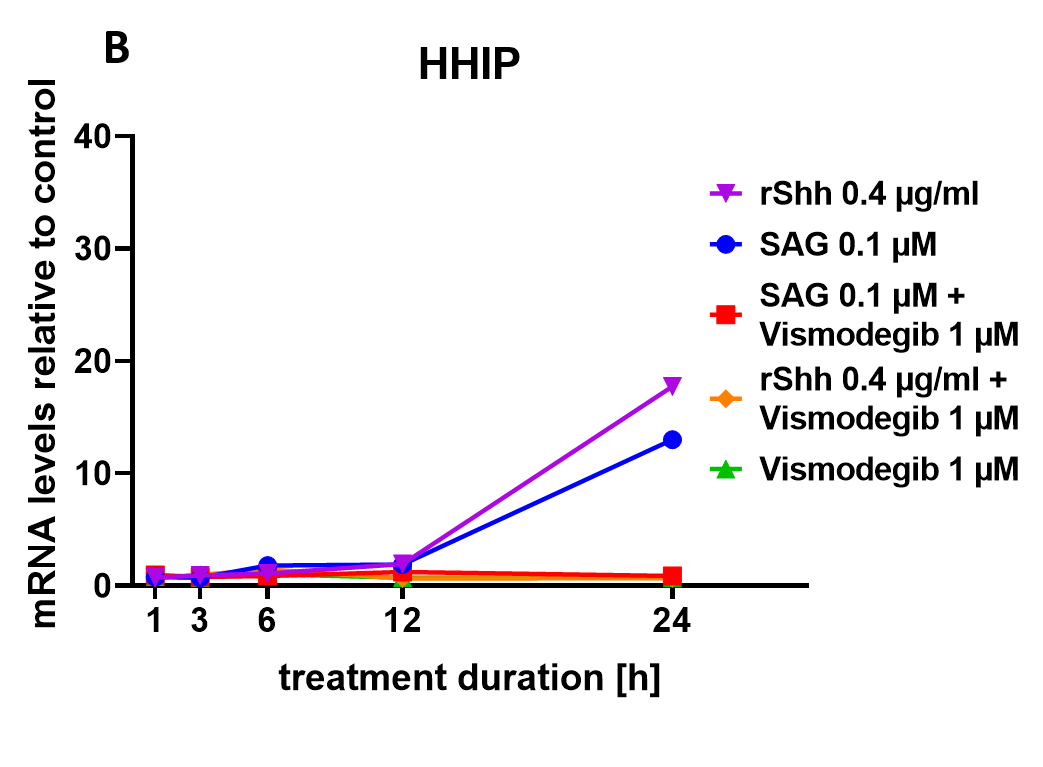

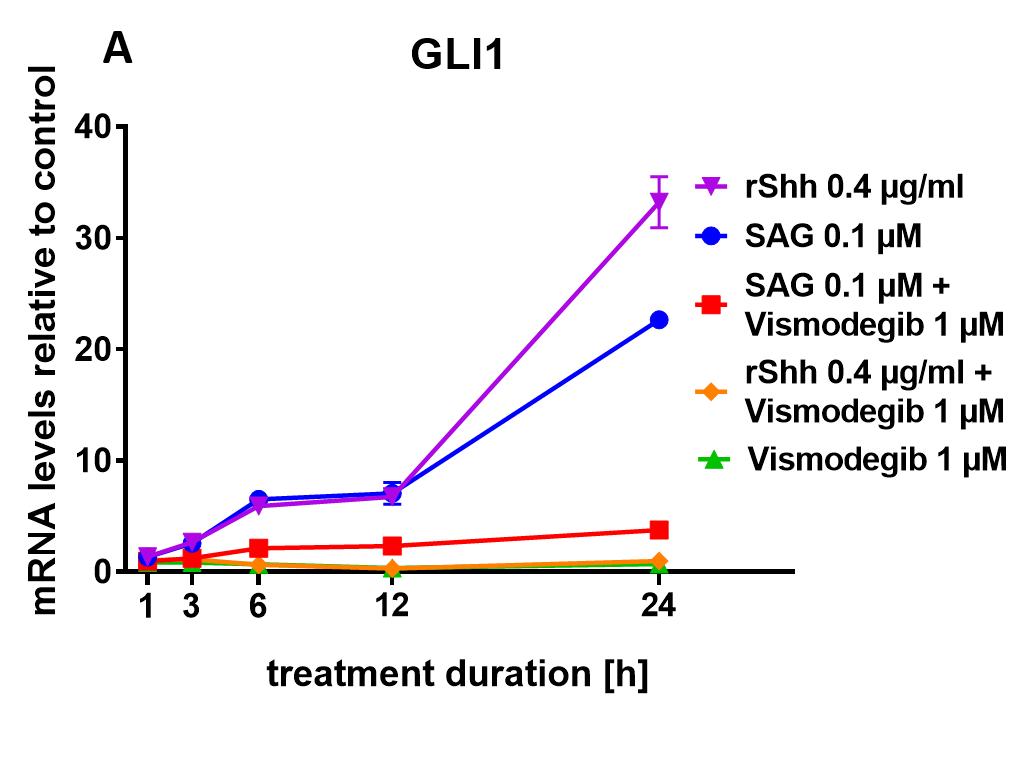


**Figure S-3.** **Distribution of protein groups identified after 5.0 min treatment (A) and 15 min treatment (B).** Intensity values of each channel of the proteome were normalized by the median of each channel. Ratios were determined by the mean of the treatment SAG or vismodegib (N=3) divided by the mean of DMSO (N=3) of the respective time point. SAG/DMSO (green graph) represents the distribution of the protein ratios of SAG treatment compared with DMSO treatment, whereas Vismo/DMSO (orange graph) describes the distribution of protein ratio of vismodegib treatment compared to control (DMSO) treatment. SAG/Vismo (blue graph) describes the phosphopeptide ratio generated by division of SAG/DMSO by Vismo/DMSO.


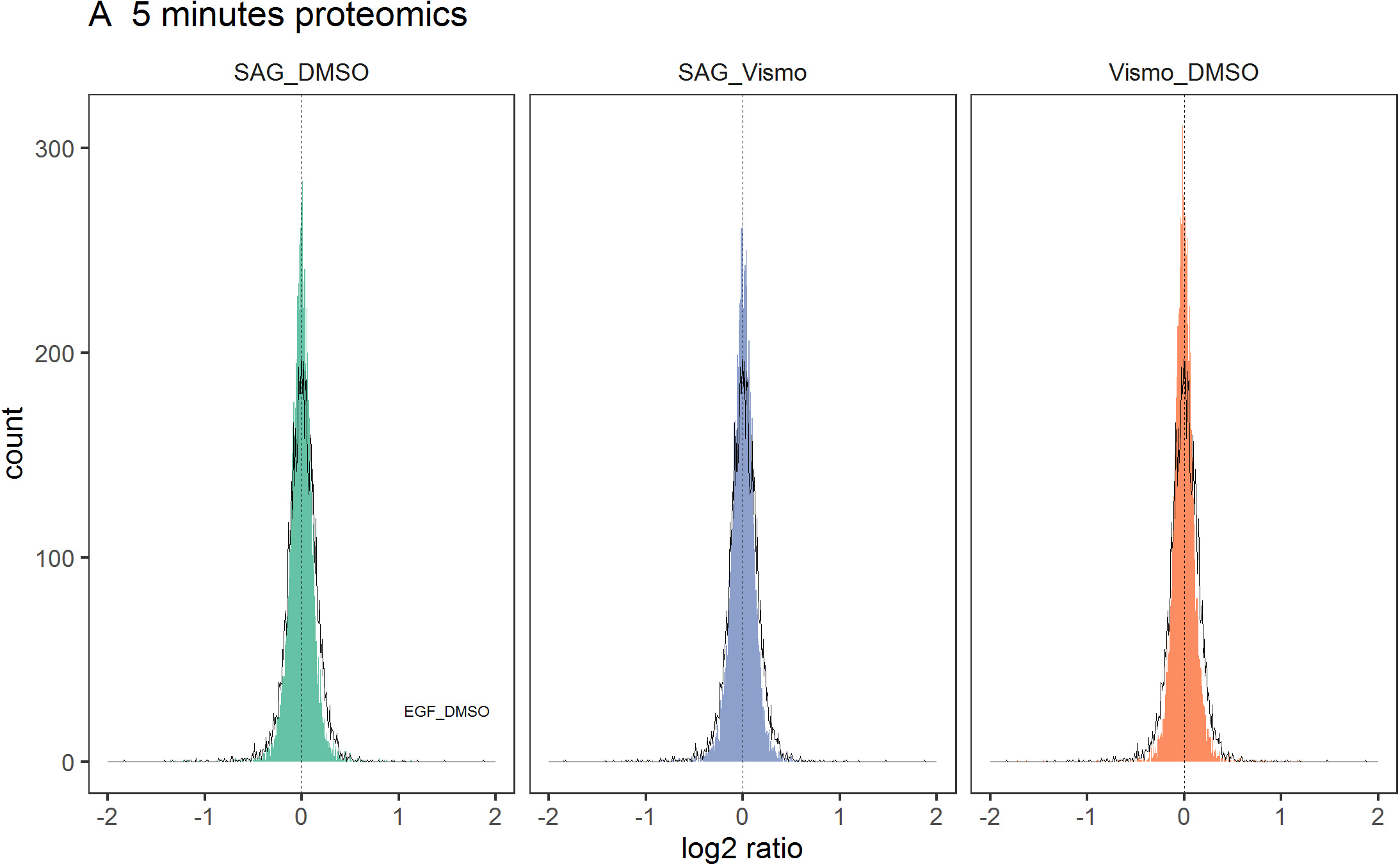

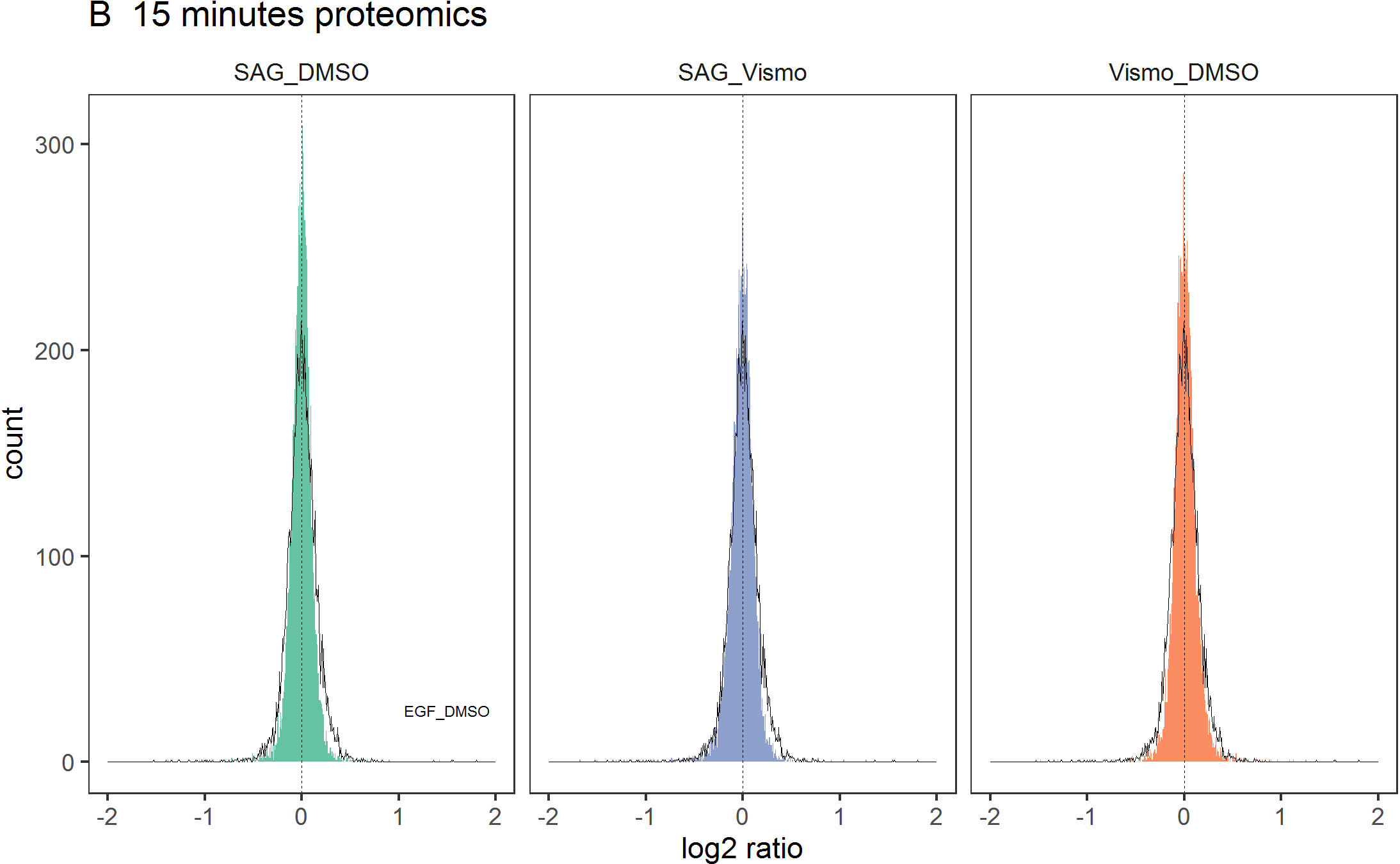


**A: 5.0 min treatment**

**B: 15 min treatment**

**SAG/DMSO**

**SAG/Vismo**

**Vismo/DMSO**

**SAG/DMSO**

**SAG/Vismo**

**Vismo/DMSO**

**Figure S-4. Ingenuity pathway analysis of all phosphopeptides identified after 15 minutes using a phosphorylation analysis.**

**A. Comparison Analysis of regulated pathways after SAG and Vismodegib treatment.**

Pathways were manually filtered for cancer relevant pathways. A log10 p-value cutoff was set to 1.3. Activated pathways are shown in orange, inhibited pathways are shown in blue. Color coding refers to the ingenuity pathway database predicted z-Scores after respective treatment.

**B. Top 10 significantly enriched pathways after 15 minutes SAG treatment (upper bar chart) and Vismodegib treatment (lower bar chart).**

The total number of proteins which are part of a particular pathway are displayed on top of the particular bar. The percentage of identified proteins is shown by the height of the bar. Upregulated phosphoproteins are displayed in orange, downregulated ones are shown in blue. The grey dots refer to the – log p-value determined by Fisher’s exact testing.


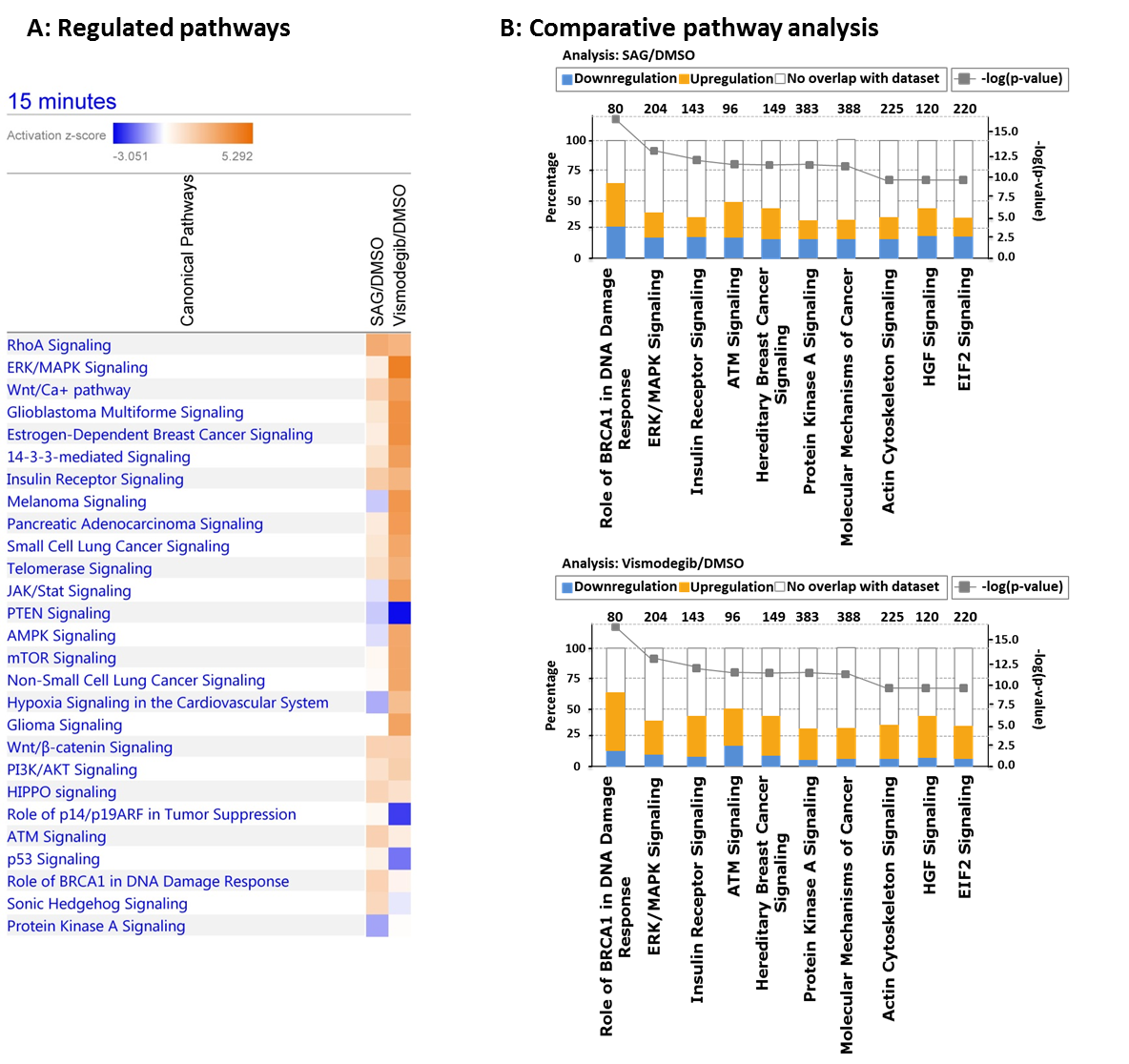


**Figure S-5. Time dependent Ingenuity pathway analysis of cancer associated pathways.** Phosphopeptide ratios of SAG/DMSO or vismodegib/DMSO determined after 15 min treatment were divided by the phosphopeptide ratio determined after 5 minutes. SAG 15min/5min reflects the phosphopeptide ratio determined after SAG treatment, while vismo 15min/5min reflects the phosphopeptide ratio determined after vismodegib treatment. Pathways were manually filtered for cancer relevant pathways. A log10 p-value cutoff was set to 1.3. Activated pathways are shown in orange, inhibited pathways are shown in blue. Color coding refers to the log2 ratio of identified phosphopeptides after respective treatment compared to DMSO as control treatment.


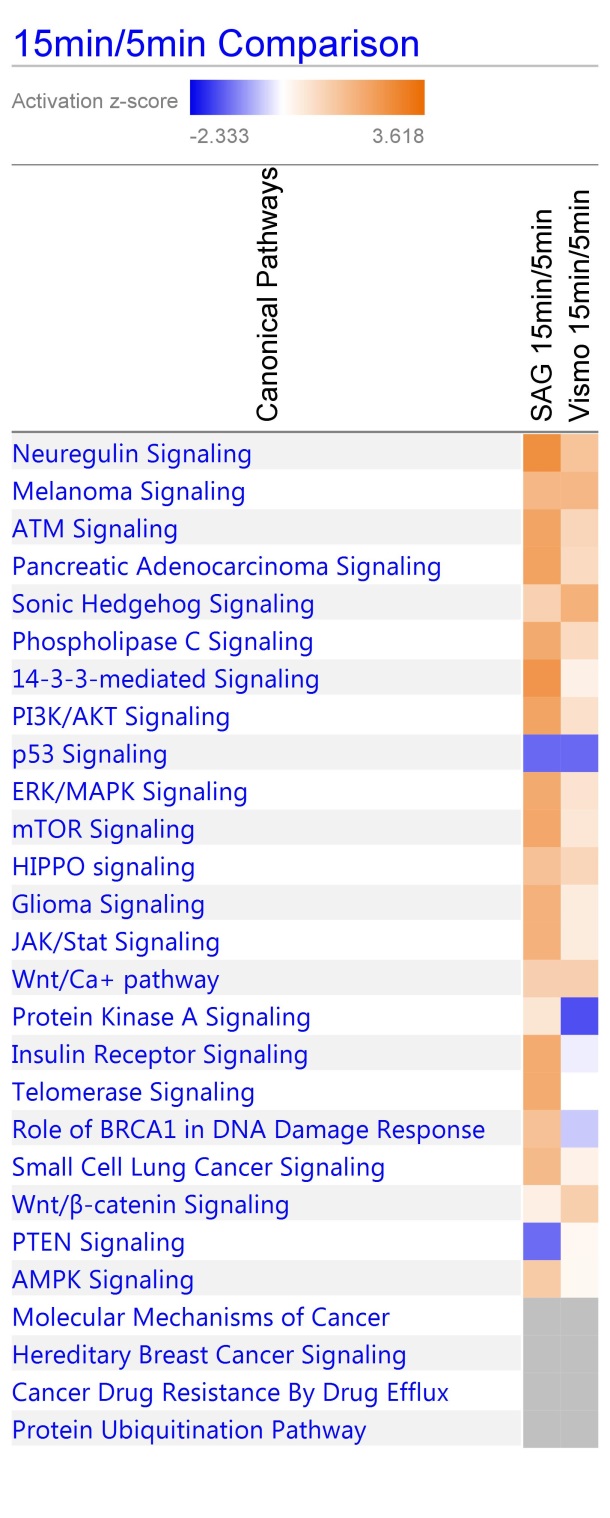


**Figure S-6.**  **Western blot validation of PKA activity modulation in response to SMO agonist and antagonists**


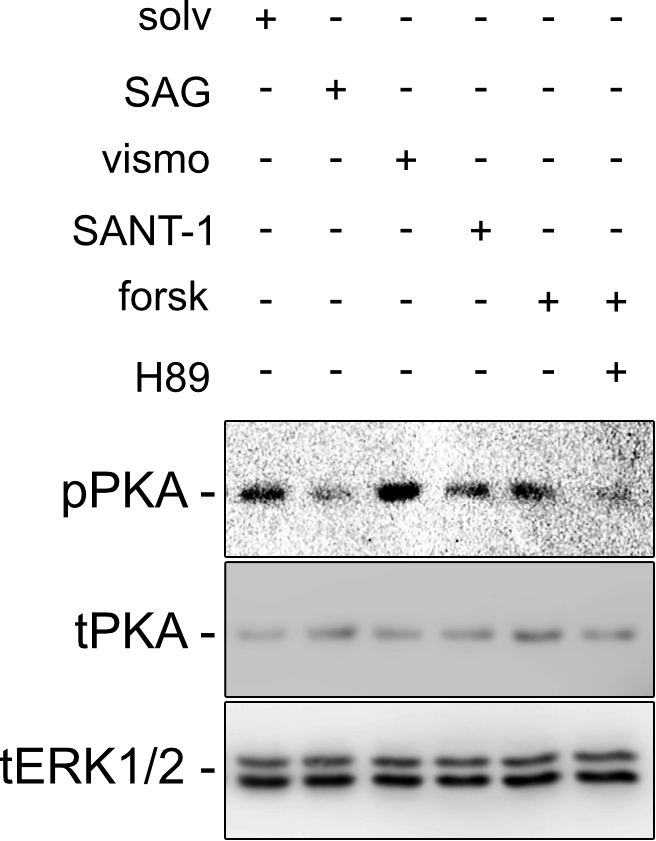


DAOY cells were cultured as described above (see Fig. S-2) and short-term treated with the compounds indicated. PKA phosphorylation was analyzed by anti-phosho-PKA (pPKA) antibody detecting active PKA phosphorylated at T197. total PKA (tPKA) and total ERK1/2 protein levels served as loading controls. solv: solvent only control (DMSO), vismo: vismodegib, forsk: forskolin, SANT-1: Smoothened antagonist-1; In line with our phopho-proteomics data, treatment with SAG resulted in reduced PKA phosphorylation, while vismodegib treatment increased phosphorylation and activation of PKA, respectively. Note that although short-term treatment with the PKA agonist forskolin did not increase pPKA levels, treatment with the PKA antagonist H89 markedly decreased PKA activation levels, demonstrating the specificity of the anti-pPKA antibody as read-out of PKA activity.

| **Table S-4** | Antibodies used for Western blot analysis | | | |
| --- | --- | --- | --- | --- |
| **Primary Antibodies** | **Species** | **Dilution** | **Company** | **Product-ID** |
| Phospho-PKA C (Thr197) Antibody | Rabbit | 1:1000 | Cell Signaling | #4781 |
| PKA C-α Antibody | Rabbit | 1:1000 | Cell Signaling | #4782 |
| p44/42 MAPK (Erk1/2) Antibody | Rabbit | 1:1000 | Cell Signaling | #9102 |
| **Secondary Antibody** |  |  |  |  |
| Anti-rabbit IgG, HRP-linked Antibody | Goat | 1:3000 | Cell Signaling | #7074 |

**Figure S-7. Overlap of quantified phosphopeptides after 5.0 and 15 min.** Venn Diagram Plotter was used to generate the Venn diagram.


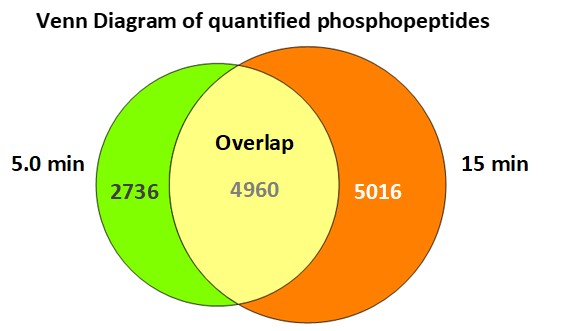


**Figure S-8. Volcano plots of phosphopeptides identified after 5.0 minutes treatment.**

**A. Distribution and significantly regulated phosphosites between SAG and DMSO treatment**.

The ratio SAG/DMSO was determined by division of the mean of proteome normalized intensity values of SAG treatment divided by the mean of proteome normalized intensity values of DMSO treatment. Limma statistical testing was applied to determine p-values and statistically significant regulated phosphosites; the negative log of the p-values is displayed on the y-axis, the log 2 ratio SAG/Vismodegib is displayed on the x-axis. Significantly down-regulated phosphosites according to a threshold of p<0.05.0 (horizontal red dashed line) are represented by orange dots; up-regulated phosphosites are represented by blue dots. Highly regulated phosphosites are indicated by Genename_phosphosite. The fold change cut-off was set to 0.5.0 and – 0.5.0, indicated by a vertical blue dashed line.

**B. Distribution and significantly regulated phosphosites between Vismodegib and DMSO treatment.**

The ratio Vismo/DMSO was determined by division of the mean of proteome normalized intensity values of Vismodegib treatment divided by the mean of proteome normalized intensity values of DMSO treatment. Limma statistical testing was applied to determine p-values and statistically significant regulated phosphosites; the negative log of the p-values is displayed on the y-axis, the log 2 ratio SAG/Vismodegib is displayed on the x-axis. Significantly down-regulated phosphosites according to a threshold of p<0.005 (horizontal red dashed line) are represented by orange dots; up-regulated phosphosites are represented by blue dots. Highly regulated phosphosites are indicated by Genename_phosphosite. The fold change cut-off was set to 0.5 and – 0.5, indicated by a vertical blue dashed line.

A

B


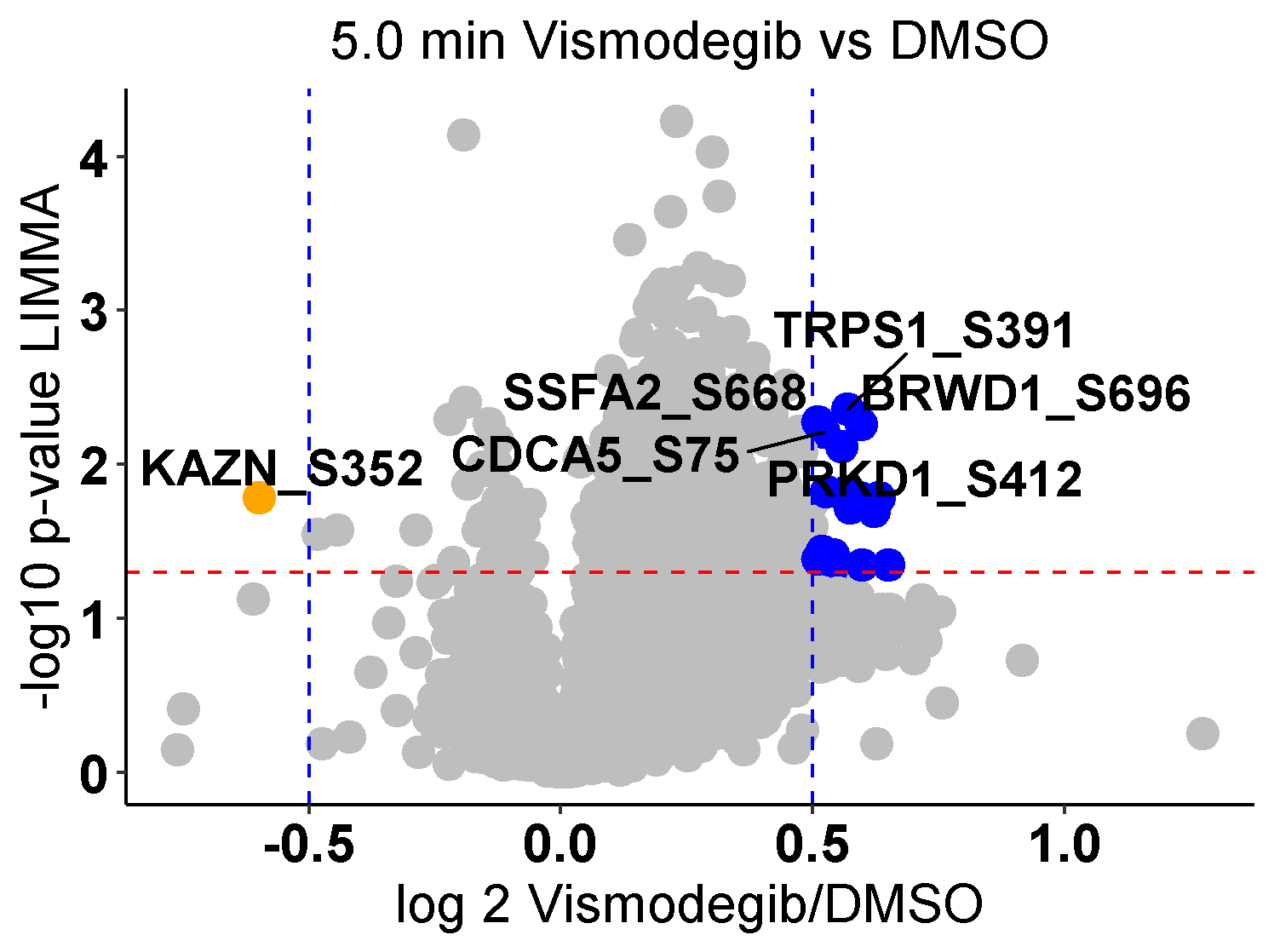

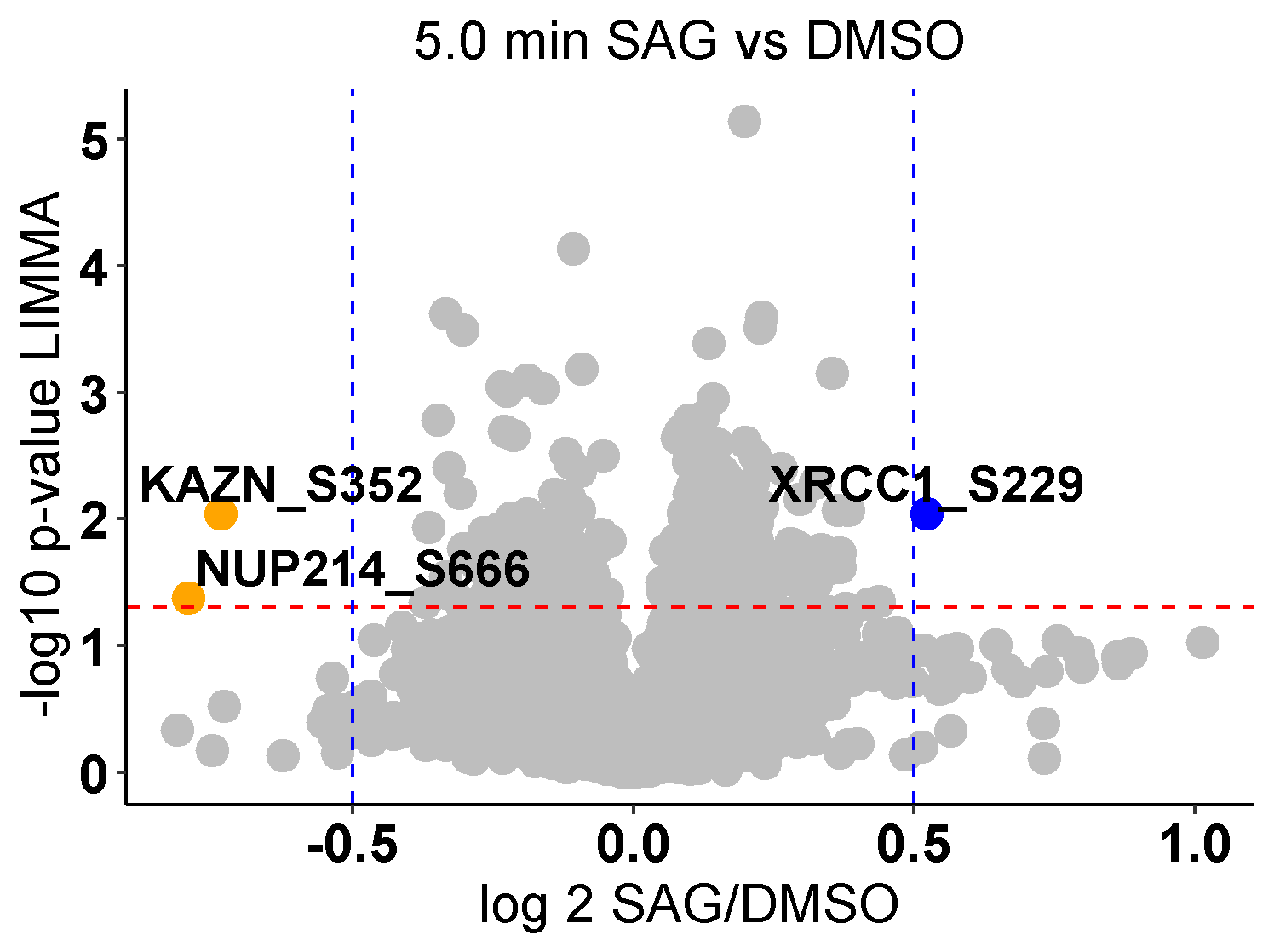


A

**Figure S-9. Kinase substrate enrichment analysis (KSEA) was performed for phosphopeptides identified after 5.0 minutes treatment for the ratio SAG/Vismo using the online platform KSEA App (casecpb.shinyapps.io/ksea/).** The ratio SAG/Vismo was determined by division of the ratio SAG_DMSO by the ratio Vismo_DMSO. The ratio SAG/Vismo was taken to infer the kinase activation score with a p-value cutoff of 0.05. All phosphosites with p-values derived by LIMMA significance testing were considered. The kinase z-score determined by the KSEA App is presented at the x-axis. Significantly activated kinases are presented in red, significantly downregulated kinases are presented in blue. The length of the bars reflects the enrichment score.


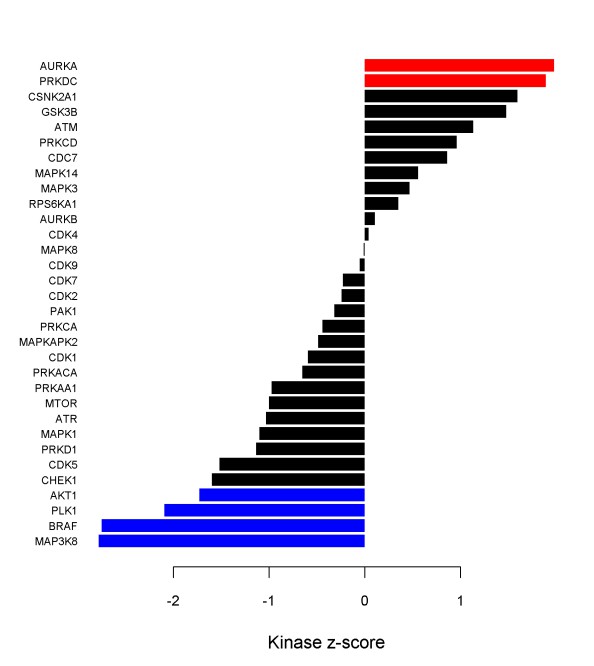


5.0 minutes SAG vs Vismodegib

**Figure S-10. Volcano plots and kinase set enrichment analysis for phosphopeptides identified after 15 minutes treatment.**

**A. Distribution and significantly regulated phosphosites between SAG and DMSO treatment**.

The ratio SAG/DMSO was determined by division of the mean of proteome normalized intensity values of SAG treatment divided by the mean of proteome normalized intensity values of DMSO treatment. Limma statistical testing was applied to determine p-values and statistically significant regulated phosphosites; the negative log of the p-values is displayed on the y-axis, the log 2 ratio SAG/Vismodegib is displayed on the x-axis. Significantly down-regulated phosphosites according to a threshold of p<0.05 (horizontal red dashed line) are represented by orange dots; up-regulated phosphosites are represented by blue dots. Highly regulated phosphosites are indicated by Genename_phosphosite. The fold change cut-off was set to 0.5 and – 0.5, indicated by a vertical blue dashed line.

**B. Distribution and significantly regulated phosphosites between Vismodegib and DMSO treatment.**

The ratio Vismo/DMSO was determined by division of the mean of proteome normalized intensity values of Vismodegib treatment divided by the mean of proteome normalized intensity values of DMSO treatment. Limma statistical testing was applied to determine p-values and statistically significant regulated phosphosites; the negative log of the p-values is displayed on the y-axis, the log 2 ratio SAG/Vismodegib is displayed on the x-axis. Significantly down-regulated phosphosites according to a threshold of p<0.05 (horizontal red dashed line) are represented by orange dots; up-regulated phosphosites are represented by blue dots. Highly regulated phosphosites are indicated by Genename_phosphosite. The fold change cutoff was set to 0.5 and – 0.5, indicated by a vertical blue dashed line.

**C. Kinase substrate enrichment analysis (KSEA) was performed using the online platform KSEA App (casecpb.shinyapps.io/ksea/).** The ratios SAG/Vismo was determined by division of the ratio SAG_DMSO by the ratio Vismo_DMSO. The ratio SAG/Vismo was taken to infer the kinase activation score with a p-value cutoff of 0.05. All phosphosites with p-values derived by LIMMA significance testing were considered. The kinase z-score determined by the KSEA App is presented at the x-axis. Significantly activated kinases are presented in red, significantly downregulated kinases are presented in blue. The length of the bars reflects the enrichment score.


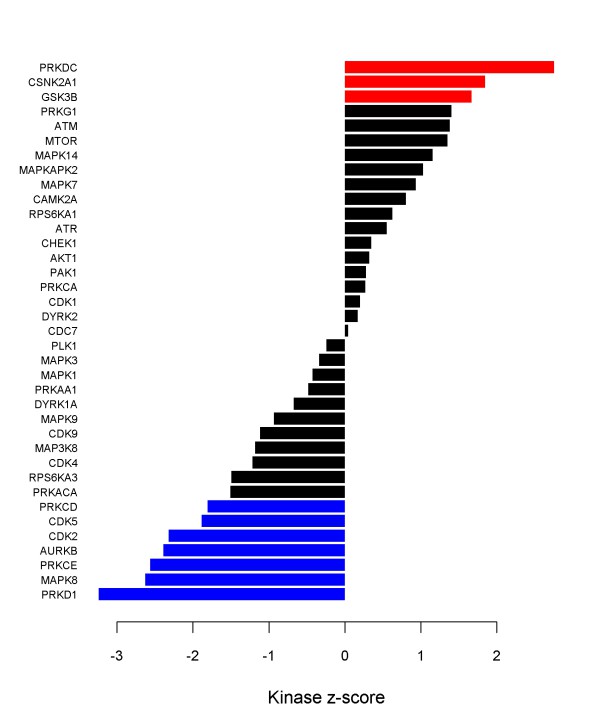

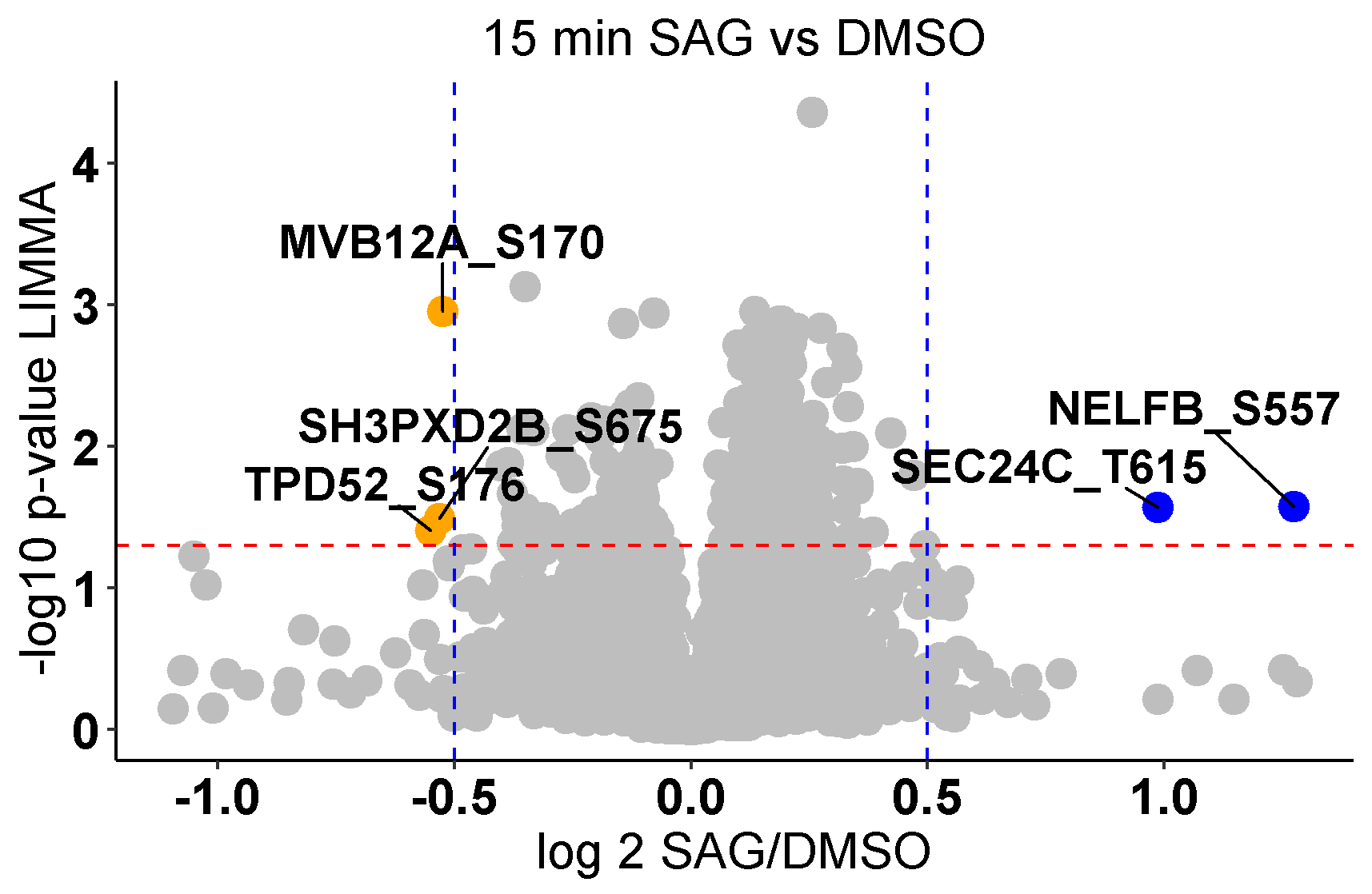

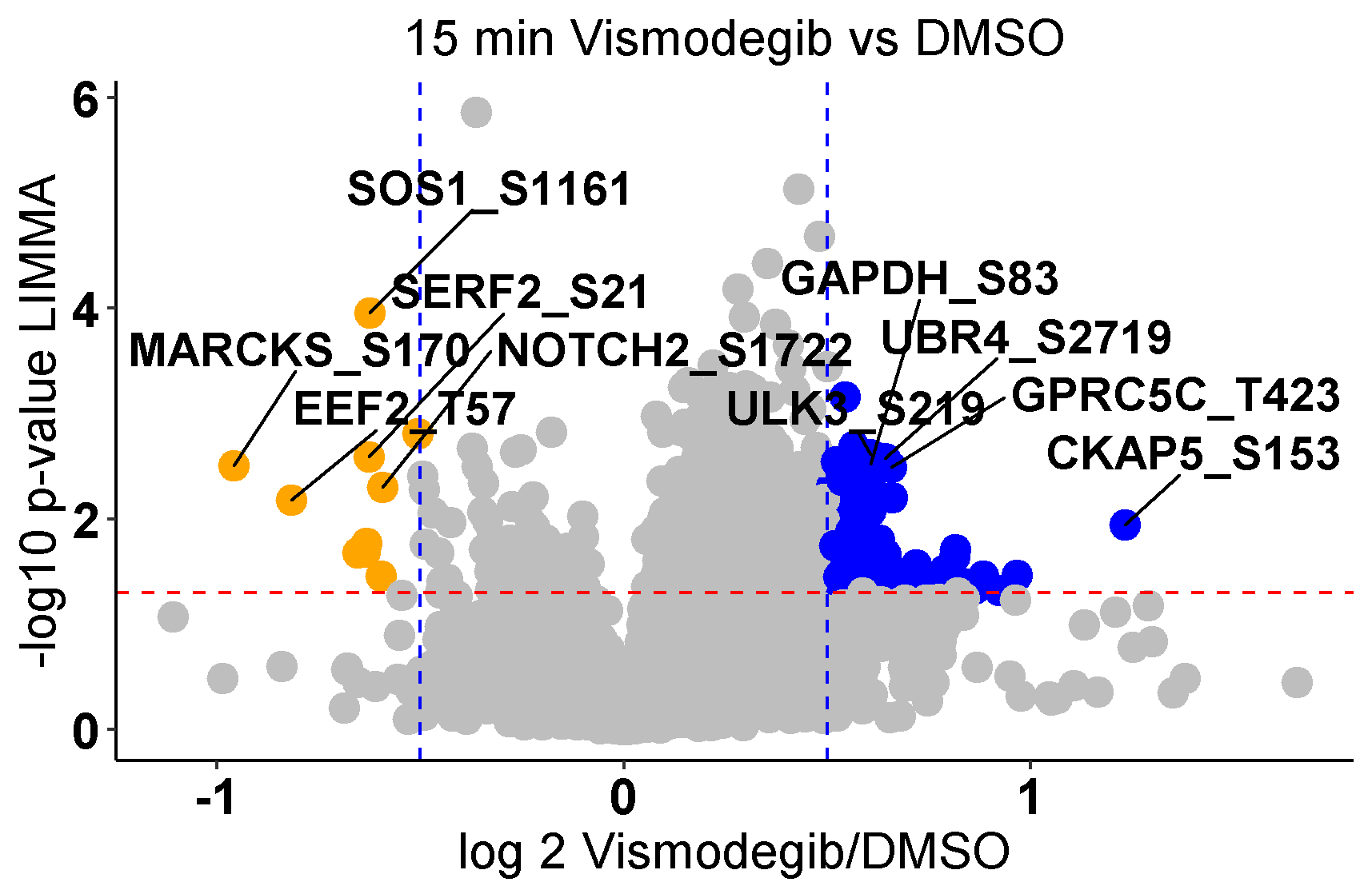


**A**

**B**

**C**

**Figure S-11. IFT172 phosphorylation and expression after 5.0 and 15 min.** The time course of change in the phosphorylation of IFT172 at Serine 273 after SAG treatment is shown in dark blue (SAG PPTX) and in red after vismodegib treatment (Vismo PPTX). Changes in the protein expression of IFT172 is shown in bright blue (SAG PTX) and dark red (Vismo PTX). PPTX refers to phosphoproteomics, PTX refers to proteomics. Graph Pad Prism Version 8.0.2 was used for visualization.

References:

Sternberg, C. *et al.* Synergistic cross-talk of hedgehog and interleukin-6 signaling drives growth of basal cell carcinoma. *International Journal of Cancer* **143**, 2943–2954 (2018).
